# A new platform for the synthesis of diketopyrrolopyrrole derivatives via nucleophilic aromatic substitution reactions

**DOI:** 10.3762/bjoc.20.169

**Published:** 2024-08-08

**Authors:** Vitor A S Almodovar, Augusto C Tomé

**Affiliations:** 1 LAQV-REQUIMTE, Department of Chemistry, University of Aveiro, 3810-193 Aveiro, Portugal

**Keywords:** diketopyrrolopyrrole, fluorescent dye, nucleophilic aromatic substitution, phenol, thiol

## Abstract

Diketopyrrolopyrroles (DPPs) are a versatile group of dyes and pigments with valuable optoelectronic properties. In this work we report the synthesis of highly fluorescent DPP derivatives through straightforward nucleophilic aromatic substitution reactions with thiols and phenols. These nucleophilic substitutions occur at room temperature and manifest a remarkable selectivity for the 4-position of the pentafluorophenyl groups. Both symmetrical (disubstitution) and non-symmetrical (monosubstitution) DPP derivatives are formed in excellent overall yields. The optical properties of the newly synthesized compounds are also discussed. The new platform may be useful for bioorthogonal chemistry.

## Introduction

Diketopyrrolopyrroles (DPPs) are a class of organic pigments discovered by serendipity in the 1970s [1–2]. Generally, N-unsubstituted DPP derivatives exhibit high melting points, low solubility in most solvents, and strong absorption in the visible region [3–4]. In turn, N-substituted DPP derivatives are soluble in common organic solvents, exhibit large molar extinction coefficients, Stokes shifts in the range of 10–70 nm and high fluorescence quantum yields [5–7].

Due to their outstanding photophysical properties, DPP-based dyes have been used in a wide range of applications, namely as organic semiconductors [8], acceptors for organic solar cells [9–10], as fluorescent probes [11–13], or as photosensitizers for photodynamic therapy and antimicrobial photodynamic therapy [14–17]. DPP derivatives with improved performance or novel properties can be prepared by conventional chemical modifications of simple DPP derivatives [3,18]. The most frequently used transformations include: i) N-alkylation with adequately functionalized alkyl groups [19–22], ii) N-arylation [23–25], and functionalization at the 3,6-di(het)aryl groups via Suzuki–Miyaura [26–28] or Sonogashira [29–31] reactions.

In this study, we report a straightforward method to obtain a diverse array of N-substituted DPP derivatives through a two-step process. Firstly, the N-alkylation of Pigment Red 254 (DPP **1**) is achieved using pentafluorobenzyl bromide, followed by a nucleophilic aromatic substitution (S_N_Ar) with thiols and phenols. This approach is based on the well-established reactivity of perfluoroaromatic compounds in nucleophilic aromatic substitutions [32–35]. By varying the reaction conditions and the number of equivalents of the nucleophile, it is possible to promote the substitution of one or more fluorine atoms. Nucleophilic substitution of fluorine atoms often necessitates harsh conditions such as elevated temperatures, strong bases, or strong nucleophiles, but our findings demonstrate that this process can be conducted under remarkably mild conditions.

## Results and Discussion

**Scheme 1:**
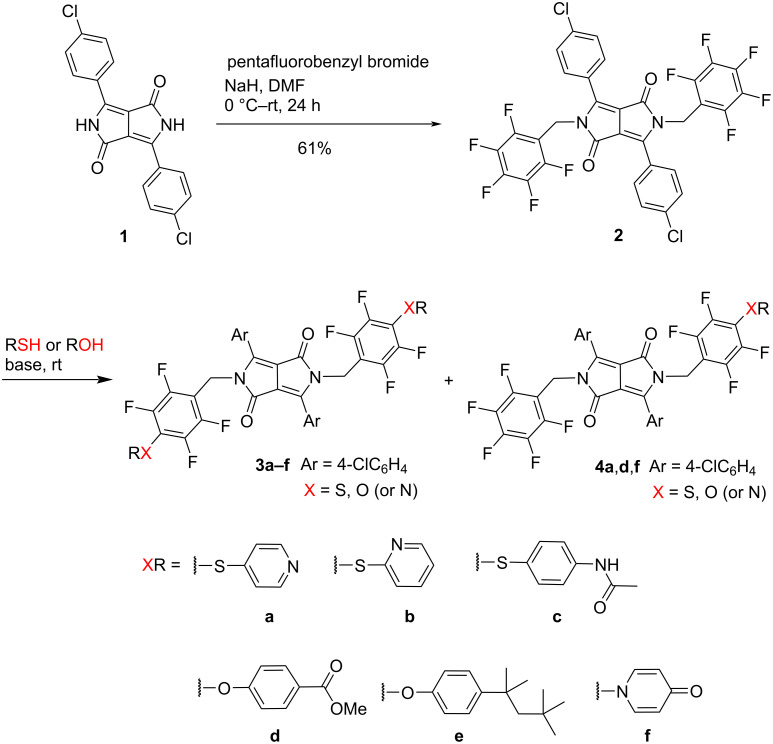
Synthesis of new diketopyrrolopyrroles via nucleophilic aromatic substitution.

The initial step of our method involved the N-alkylation of DPP **1** with pentafluorobenzyl bromide (Scheme 1). Although a similar reaction had been previously reported for other DPP derivatives, the experimental conditions used (DMF, K_2_CO_3_, 120 °C, 2 h) resulted in very low yields (6–16%) for the formation of *N*,*N’*-bis(pentafluorobenzyl)-DPP derivatives [36]. Changing the base to NaH and performing the reaction at a lower temperature, enabled to obtain DPP **2** in a reasonable yield (61%) and allowed us to use it as a starting material for generating new DPP derivatives through nucleophilic aromatic substitution reactions with thiols and phenols.

The main objective of this study was to employ the *N,N’*-bis(pentafluorobenzyl)-DPP **2** as an electrophile and investigate its reactivity with thiols and phenols (Scheme 1). All S_N_Ar reactions were performed in dry DMF at room temperature, in the presence of a base (K_2_CO_3_ or Cs_2_CO_3_). Room temperature was chosen due to the observed rapid degradation of the starting material at elevated temperatures. The work described herein allowed us to assess the potential of DPP **2** as a novel platform for obtaining functionalized DPP derivatives. As anticipated, it displayed reactivity with thiols and phenols through nucleophilic aromatic substitution at the pentafluorobenzyl groups, yielding both symmetrical (disubstitution) and non-symmetrical (monosubstitution) derivatives in satisfactory yields (Scheme 1).

Thiols are excellent nucleophiles and generally react under mild conditions, resulting in the substitution of the 4-F atom of the pentafluorophenyl groups. In this case, reactions with thiols were performed in dry DMF and K_2_CO_3_ was used as the base. Three different thiols were tested: pyridine-4-thiol, pyridine-2-thiol and 4-(acetylamino)benzenethiol. The reaction with pyridine-4-thiol yielded a mixture of the di- and monosubstituted compounds **3a** and **4a** in 51% and 23% yields, respectively. Conversely, for the reaction with pyridine-2-thiol, exclusively produced the disubstituted compound **3b** in an 85% yield. Furthermore, the reaction with 4-(acetylamino)benzenethiol led to the selective formation of the disubstituted compound **3c** in 53% yield.

Phenols are less nucleophilic than thiols and, depending on the substitution pattern, a stronger base is often required to generate the corresponding alkoxide, which is the effective nucleophile. So, in this case, Cs_2_CO_3_ was employed as the base. The reaction of DPP **2** with methyl 4-hydroxybenzoate yielded compounds **3d** and **4d** in 56% and 14% yield, respectively. When reacting with 4-(2,4,4-trimethylpentan-2-yl)phenol, the disubstituted compound **3e** was obtained in 63% yield. In contrast to the reaction with pyridine-4-thiol, which resulted in the S-substituted product **3a**, the reaction with 4-hydroxypyridine led exclusively to the formation of the pyridin-4-one-derived compounds **3f** and **4f,** in 45% and 13% yield, respectively. The substitution occurred at the nitrogen atom rather than the oxygen due to the preferential existence of 4-hydroxypyridine in the pyridin-4-one tautomeric form [37–39]. The structures of dyes **3a**–**f**, **4a**, **4d** and **4f** were unambiguously established through their ^1^H, ^13^C and ^19^F NMR and mass spectra.

The ^1^H NMR spectra of the symmetrical compounds displayed a characteristic signal for the N–CH_2_ protons as a singlet at approximately δ 5.10 ppm. Signals of the 4-chlorophenyl groups appeared as AB systems centred at around δ 7.9 ppm. For the non-symmetrical derivatives, two singlets were observed at approximately δ 5.05 and 5.10 ppm, corresponding to the protons of the N–CH_2_C_6_F_5_ and N–CH_2_C_6_F_4_XR groups, respectively. All ^19^F NMR spectra confirmed the selective substitution of the 4-fluorine atoms (in one or in two rings) by the disappearance of the signal corresponding to the resonance of those atoms. Mass spectra of compounds **3a–f**, **4a**, **4d** and **4f** consistently displayed the protonated molecular ion [M + H]^+^ as the base peak.

**Figure 1:**
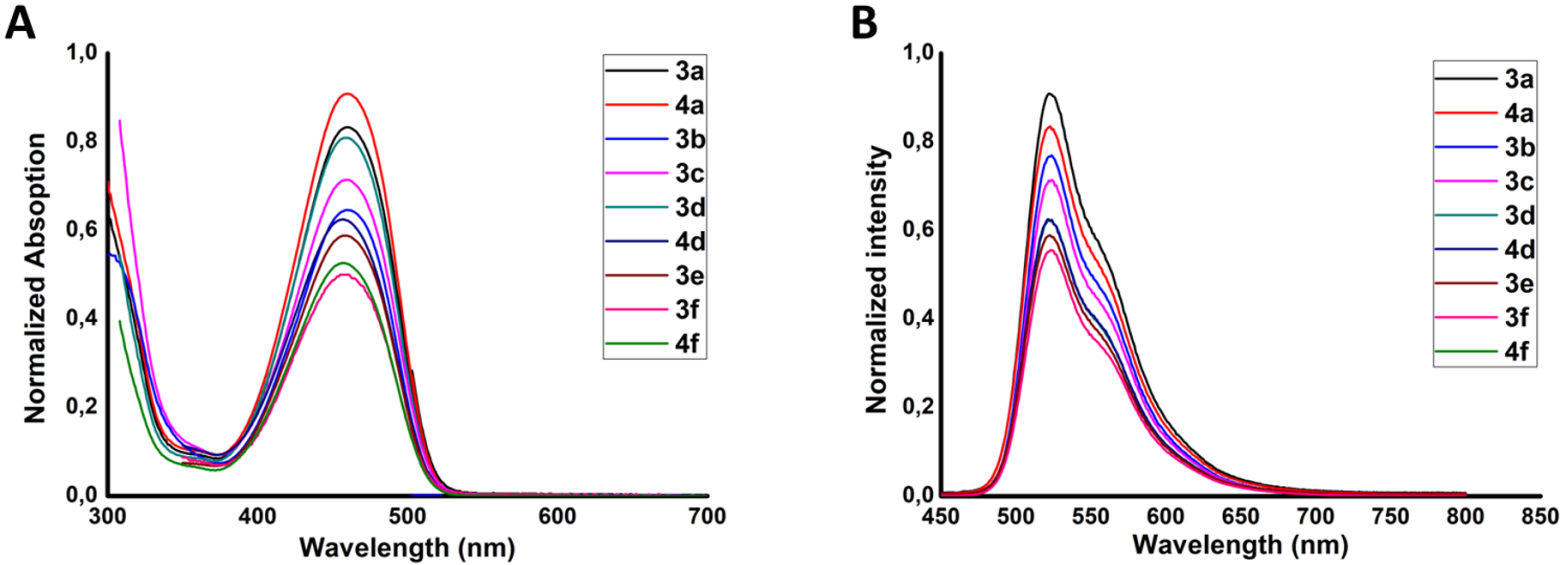
(A) Absorption and (B) fluorescence spectra of compounds **3a–f**, **4a**, **4d** and **4f**, in DMF. Different concentrations of the compounds were used to allow visualization of each spectrum.

The UV–vis and fluorescence spectra of DPP derivatives **3a–f**, **4a**, **4d** and **4f** in DMF are presented in Figure 1, and their photophysical properties are summarized in Table 1. These compounds are highly fluorescent, and their UV–vis spectra are very similar. These results indicate that substituents with different functional groups can be attached to DPP **2** without significant modification of their optical properties. The observed Stokes shifts for dyes **3** and **4** averaged in the range of 60–70 nm. All compounds exhibited high fluorescence quantum yields, ranging from 0.66 to 0.83, confirming their potential applications in fluorescence imaging, sensors, and optoelectronic devices. A comprehensive discussion of the potential uses of these fluorescent substances in areas such as materials science, biology, or chemistry may provide a deeper understanding of their significance.

**Table 1:** Spectroscopic data for the new compounds (between 1 × 10^–6^ M and 4 × 10^–5^ M in DMF).

Compound	Absorption λ_max_ (nm)	Log ε (M^–1^ cm^–1^)	Emission λ_max_ (nm)	Stokes shift (cm^–1^)	Ф_F_ (DMF)^a^

**3a**	460	4.15	521	2545	0.69
**4a**	460	4.11	522	2582	0.68
**3b**	461	4.17	524	2608	0.83
**3c**	461	4.25	525	2644	0.78
**3d**	457	4.43	522	2687	0.73
**4d**	459	4.46	521	2593	0.72
**3e**	460	4.03	522	2547	0.71
**3f**	456	4.33	523	2809	0.83
**4f**	458	4.32	524	2750	0.66

^a^Excitation at 436 nm. *N,N’*-Dibenzyl-DPP was used as the fluorescence quantum yield reference: Φ_F_ = 0.88, in chloroform [40].

## Conclusion

In conclusion, novel DPP derivatives were synthesized through the reaction of a *N*,*N*’-bis(pentafluorobenzyl)-DPP with thiols and phenols. The nucleophilic aromatic substitution reactions took place under exceptionally mild experimental conditions, and the resulting compounds were isolated in reasonable yields. The newly synthesized compounds display high fluorescence quantum yields and moderate Stokes shifts, which are crucial attributes for their potential application in diverse fields, particularly in biological or technical applications. Additionally, it is crucial to highlight the chemical versatility of compound **2**, which allows the attachment of various functional units without significantly altering its optical properties. This versatility holds significant promise in the design and synthesis of innovative molecules tailored for specific purposes. This study not only contributes to the expansion of accessible N-substituted DPP derivatives but also reveals that such transformations can be achieved with outstanding efficiency and environmental sensitivity by employing mild reaction conditions.

## Experimental

### Chemicals and instrumentation

The reagents used in this work were purchased from Merck Life Science (Algés, Portugal) or TCI Europe N.V. (Belgium) and were used as received. Pigment Red 254 was purchased from TCI Europe N.V. The solvents were used as received or distilled and dried by standard procedures. Analytical thin-layer chromatography (TLC) was carried out on precoated sheets with silica gel (Merck 60, 0.2 mm thick). Preparative TLC was carried out on 20 cm × 20 cm glass plates precoated with a layer of silica gel 60 (0.5 mm thick) and activated in an oven at 100 °C for 12 h. Melting points were determined with a Büchi B-540 apparatus. NMR spectra were recorded on a Bruker DRX 300 Avance operating at 300.13 MHz (for ^1^H NMR), at 75.47 MHz (for ^13^C NMR) and 282 MHz (for ^19^F NMR). Deuterated chloroform (CDCl_3_) was used as the solvent and tetramethylsilane (TMS) as the internal reference. The chemical shifts (δ) are expressed in parts per million (ppm) and the coupling constants (*J*) in hertz (Hz). UV–vis spectra were recorded on a Shimadzu UV-2501PC spectrophotometer using DMF as the solvent. The emission spectra were recorded with a Jasco FP-8300 spectrofluorometer using DMF as the solvent. Mass spectra were recorded using a Micromass Q-TOF-2TM mass spectrometer and CHCl_3_ as the solvent. The NMR, absorption and emission spectra of the new compounds are shown in Supporting Information File 1.

### Synthesis

#### 3,6-Bis(4-chlorophenyl)-2,5-bis(pentafluorobenzyl)-2,5-dihydropyrrolo[3,4-*c*]pyrrole-1,4-dione (**2**)

A suspension of DPP **1** (1 g, 2.8 mmol) and NaH (11.2 mmol) in DMF (60 mL) was stirred at 0 °C under a nitrogen atmosphere for 30 min. At this temperature, and under vigorous stirring, a solution of pentafluorobenzyl bromide (1.7 mL, 11.2 mmol) in DMF (8 mL) was added dropwise. The mixture was stirred for 24 h at room temperature and then it was diluted with CH_2_Cl_2_ and water. The organic layer was separated and washed with water and brine. The product was isolated by column chromatography on silica gel using CH_2_Cl_2_ as the eluent. Yield: 61%; mp: 278–280 °C; ^1^H NMR (300 MHz, CDCl_3_) δ (ppm) 7.62–7.66 (m, 4H), 7.47–7.52 (m, 4H), 5.03 (s, 4H); ^13^C NMR (75 MHz, CDCl_3_) δ (ppm) 161.7, 147.2, 138.01, 129.8, 129.6, 125.6, 110.0, 29.7; ^19^F NMR (282 MHz, CDCl_3_) δ (ppm) −138.11 to −138.29 (m, 4F), −149.90 (t, *J* = 21.4 Hz, 2F), −157.63 to −157.91 (m, 4F); ESIMS *m*/*z*: 717.0 (M + H^+^, 100%).

#### General procedure for the nucleophilic aromatic substitution reactions

The reactions of DPP **2** with thiols and phenols were carried out in dry DMF, at room temperature, and in the presence of K_2_CO_3_ or Cs_2_CO_3_. Once the starting DPP was completely consumed (after 2–3 hours with thiols and 5–6 hours with phenols), the reaction mixtures were diluted with CH_2_Cl_2_ and water. The organic layer was then separated and washed with brine and water. The products were isolated by preparative TLC using CH_2_Cl_2_/hexane mixtures as the eluent.

**Compound 3a.** Yield: 51%; mp 274–276 °C; ^1^H NMR (CDCl_3_, 300 MHz) δ (ppm) 8.43 (AA’XX’, *J* = 6 Hz, 4H), 7.68 (AA’BB’, *J* = 8.7 Hz, 4H), 7.51 (AA’BB’, *J* = 8.7 Hz, 4H), 6.91 (AA’XX’, *J* = 6 Hz, 4H), 5.15 (s, 4H); ^19^F NMR (282 MHz, CDCl_3_) δ (ppm) −153.53 to −153.65 (m, 4F), −162.60 to −162.73 (m, 4F); ESIMS *m*/*z*: 899.1 (M + H^+^, 100%).

**Compound 4a.** Yield: 23%; mp 269–273 °C; ^1^H NMR (300 MHz, CDCl_3_) δ (ppm) 8.45 (AA’XX’, *J* = 6.3 Hz, 2H), 7.70–7.64 (m, 4H), 7.52 (AA’BB’, *J* = 8.7 Hz, 4H), 7.07 (AA’XX’, *J* = 6.3 Hz, 2H), 5.15 (s, 2H), 5.05 (s, 2H); ^13^C NMR (125 MHz, CDCl_3_) δ (ppm) 161.7, 149.7, 147.6, 146.95, 145.9, 138.1, 129.9, 129.6, 125.6, 121.1, 109.9, 109.7, 34.9, 34.5; ^19^F NMR (282 MHz, CDCl_3_) δ (ppm) −126.79 to −126.90 (m, 2F), –135.53 to –153.81 (m, 2F), −137.99 to −138.50 (m, 2F), −149.74 (t, *J* = 21.3 Hz, 1F), −157.60 to −157.78 (m, 2H); ESIMS *m*/*z*: 802.3 (M + H^+^, 100%).

**Compound 3b.** Yield: 85%; mp 270–272 °C; ^1^H NMR (300 MHz, CDCl_3_) δ (ppm) 8.3–8.32 (m, 2H), 7.68 (AA’BB’, *J* = 8.7 Hz, 4H), 7.55 (ddd, *J* = 8.1, 7.4, 1.9 Hz, 2H), 7.49 (AA’BB’, *J* = 8.7 Hz, 4H), 7.15–7.02 (m, 4H), 5.13 (s, 4H); ^13^C NMR (125 MHz, CDCl_3_) δ (ppm) 161.8, 155.5, 150.0, 147.3, 137.9, 137.1, 129.9, 129.5, 125.8, 121.7, 121.1, 116.4, 110.0, 35.1; ^19^F NMR (282 MHz, CDCl_3_) δ (ppm) −127.66 to −127.79 (m 4F), −138.03 to −138.25 (m, 4F); ESIMS *m/z*: 899.0 (M + H^+^, 100%).

**Compound 3c.** Yield: 53%; mp 252–256 °C; ^1^H NMR (300 MHz, DMSO-*d*_6_) δ (ppm) 10.08 (s, 2H), 7.78 (AA’BB’, *J* = 8.7 Hz, 4H), 7.55–7.61 (m, 8H), 7.22 (AA’BB’, *J* = (8.7 Hz, 4H), 5.09 (s, 4H), 2.03 (s, 6H); ^13^C NMR (125 MHz, DMSO) δ (ppm) 169.1, 164.7, 161.2, 147.3, 139.9, 136.7, 131.6, 130.9, 130.6, 129.5, 126.4, 125.4, 120.4, 120.1, 109.3, 31.3, 24.5; ^19^F NMR (282 MHz, DMSO-*d*_6_) δ (ppm) −131.31 to −131.54 (m, 4F), −137.94 to −138.07 (m, 4F); ESIMS *m/z*: 1011.0 (M + H^+^, 100%).

**Compound 3d.** Yield: 56%; mp 249–251 °C; ^1^H NMR (300 MHz, CDCl_3_) δ (ppm) 8.02 (AA’XX’, *J* = 9 Hz, 4H), 7.68 (AA’BB’, *J* = 8.7 Hz, 4H), 7.52 (AA’BB’, *J* = 8.7 Hz, 4H), 6.88 (AA’XX’, *J* = 9 Hz, 4H), 5.11 (s, 4H), 3.90 (s, 6H); ^19^F NMR (282 MHz, CDCl_3_) δ (ppm) −138.23 to −138.35 (m, 4F), −149.59 to −149.96 (m, 4F); ESIMS *m/z*: 981.0 (M + H^+^, 100%).

**Compound 4d.** Yield: 14%; mp 255–257 °C; ^1^H NMR (300 MHz, CDCl_3_) δ (ppm) 8.02 (AA’XX’, *J* = 9 Hz, 2H), 7.67–7.59 (m, 4H), 7.52–7.44 (m, 4H), 6.88 (AA’XX’, *J* = 9 Hz, 2H), 5.10 (s, 2H), 5.03 (s, 2H), 3.92 (s, 3H); ^19^F NMR (282 MHz, CDCl_3_) δ (ppm) −138.14 to −138.37 (m, 4F), −149.82 to −150.01 (m, 3F), −157.70 to −157.85 (m, 2F); ESIMS *m/z*: 849.0 (M + H^+^, 100%).

**Compound 3e.** Yield: 63%; mp 262–265 °C; ^1^H NMR (300 MHz, CDCl_3_) δ (ppm) 7.66 (AA’BB’, *J* = 8.7 Hz, 4H), 7.47 (AA’BB’, *J* = 8.7 Hz, 4H), 7.28 (AA’BB’, *J* = 9 Hz, 4H), 6.75 (AA’BB’, *J* = 9 Hz, 4H), 5.09 (s, 4H), 1.70 (s, 4H), 1.34 (s, 12H), 0.70 (s, 18H); ^13^C NMR (125 MHz, DMSO) δ (ppm) 161.7, 154.7, 147.3, 145.8, 137.9, 129.9, 129.4, 127.4, 125.8, 114.8, 109.8, 57.0, 38.2, 34.5, 32.3, 31.8, 31.6; ^19^F NMR (282 MHz, CDCl_3_) δ (ppm) −139.07 to −139.30 (m, 4F), −150.36 to −150.46 (m, 4F); ESIMS *m/z*: 1089.2 (M + H^+^, 100%).

**Compound 3f.** Yield: 45%; mp 253–255 °C; ^1^H NMR (300 MHz, CDCl_3_) δ (ppm) 7.69 (AA’BB’, *J* = 8.7 Hz, 4H), 7.55 (AA’BB’, *J* = 8.7 Hz, 4H), 7.25–7.21 (m, 4H), 6.48 (d, *J* = 8.1 Hz, 4H), 5.12 (s, 4H); ^19^F NMR (282 MHz, CDCl_3_) δ (ppm) −137.72 to −137.84 (m, 4F), −145.60 to −145.71 (m, 4F); ESIMS *m/z*: 867.1 (M + H^+^, 100%).

**Compound 4f.** Yield: 13%; mp 248–250 °C; ^1^H NMR (300 MHz, CDCl_3_) δ (ppm) 7.73–7.58 (m, 4H), 7.58–7.46 (m, 4H), 7.28–7.25 (m, 2H), 6.53 (d, *J* = 7.8 Hz, 2H), 5.10 (s, 2H), 5.04 (s, 2H); ^13^C NMR (125 MHz, DMSO) δ (ppm) 177.7, 161.2, 147.4, 141.8, 136.9, 131.1, 130.9, 129.6, 126.3, 118.0, 109.4, 34.9, 34.5; ^19^F NMR (282 MHz, CDCl_3_) δ (ppm): −135.56 to −135.69 (m, 2F), −138.23 to −138.34 (m, 2F), −144.36 to −144.49 (m, 2F), −149.67 (t, *J* = 20.8 Hz, 1F), −157.53 to −157.84 (m, 2F); ESIMS *m*/*z*: 792.1 (M + H^+^, 100%).

## Supporting Information

File 1 ^1^H NMR, ^13^C NMR and ^19^F NMR spectra; MS, UV–vis and emission spectra.

## Data Availability

All data that supports the findings of this study is available in the published article and/or the supporting information to this article.
