# Supplementary material for: A new platform for the synthesis of diketopyrrolopyrrole derivatives via nucleophilic aromatic substitution reactions
Source: Beilstein J Org Chem. 2024 Aug 8;20:1933–9. doi: 10.3762/bjoc.20.169 (PMC11318607; doi:10.3762/bjoc.20.169)

BEILSTEIN JOURNAL OF ORGANIC CHEMISTRY

Supporting Information

for

A new platform for the synthesis of diketopyrrolopyrrole derivatives via nucleophilic aromatic substitution reactions

Vitor A. S. Almodovar and Augusto C. Tomé

Beilstein J. Org. Chem. **2024,** *20,* 1933–1939. doi:10.3762/bjoc.20.169

^{1}H NMR, ^{13}C NMR and ^{19}F NMR spectra; MS, UV–vis and emission spectra

License and Terms: This is a supporting information file under the terms of the Creative Commons Attribution License (https://creativecommons.org/licenses/by/4.0). Please note that the reuse, redistribution and reproduction in particular requires that the author(s) and source are credited and that individual graphics may be subject to special legal provisions.
The license is subject to the *Beilstein Journal of Organic Chemistry* terms and conditions: (https://www.beilstein-journals.org/bjoc/terms)

Compound 2

^{1}H NMR spectrum of 2

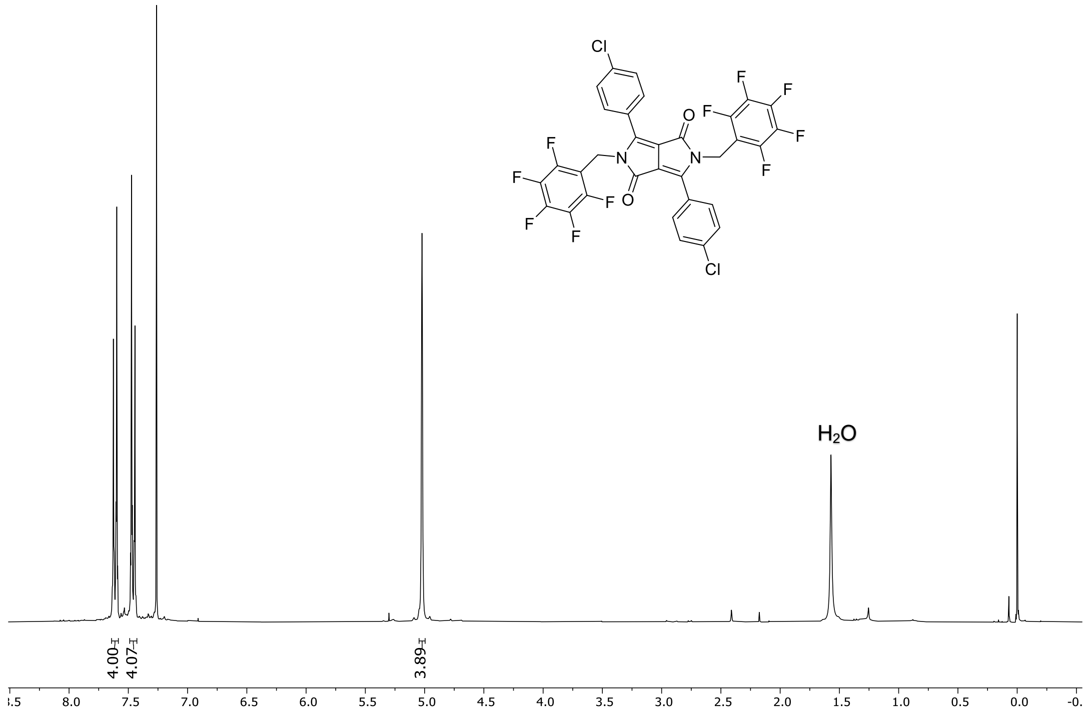

^{19}F NMR spectrum of 2

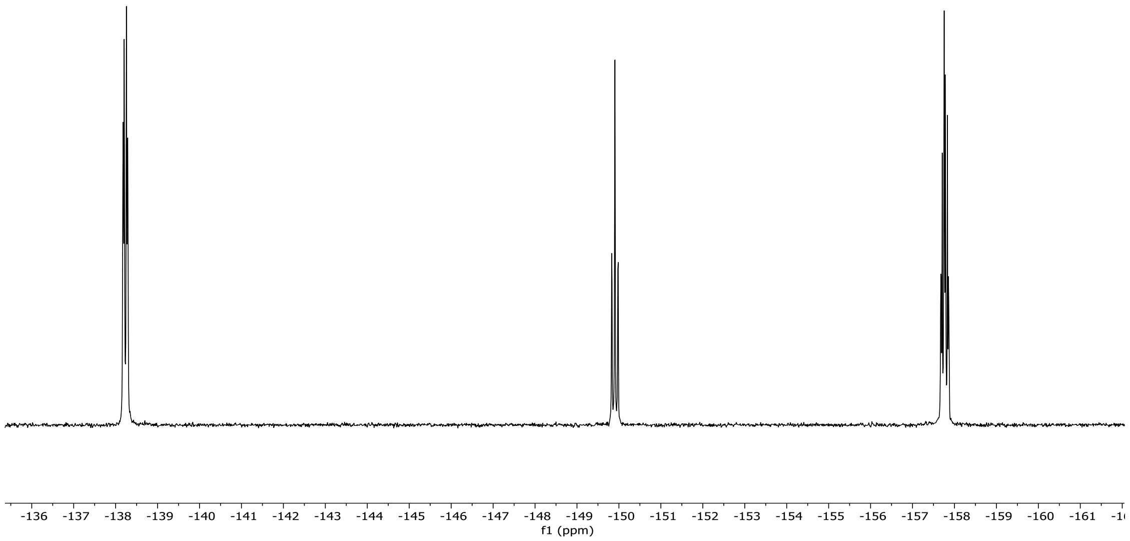

^{13}C NMR spectrum of 2

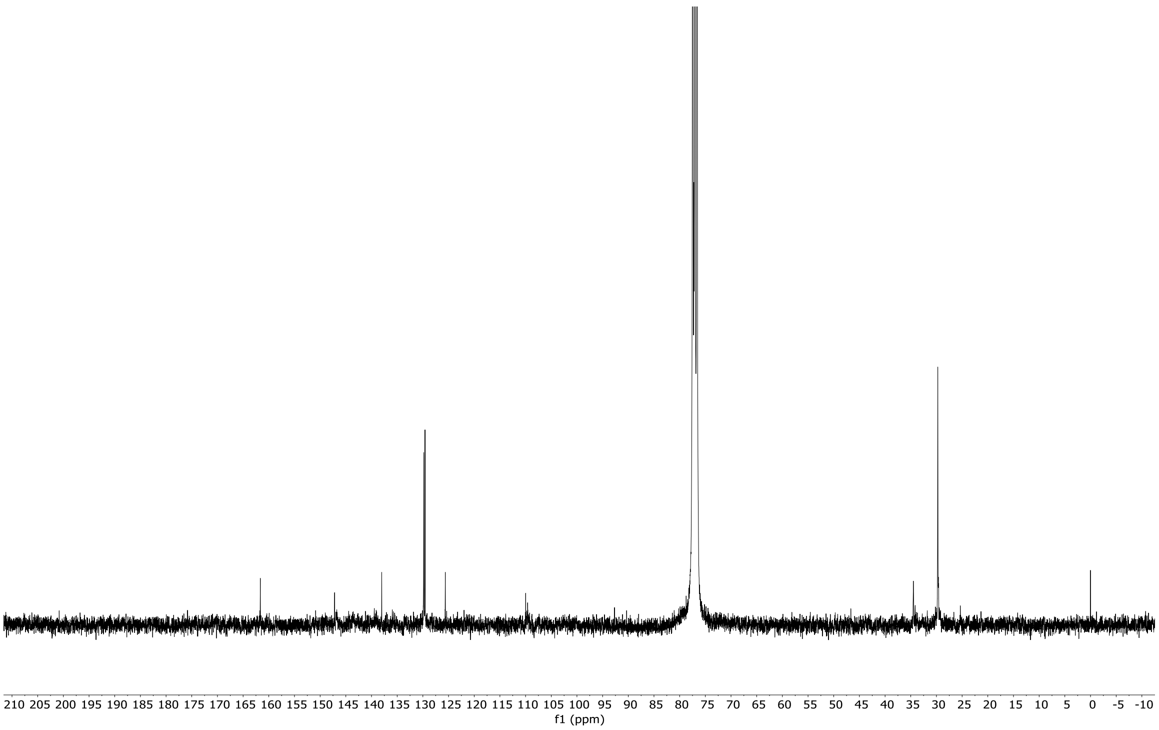

Compound 3a

^1H NMR spectrum of 3a

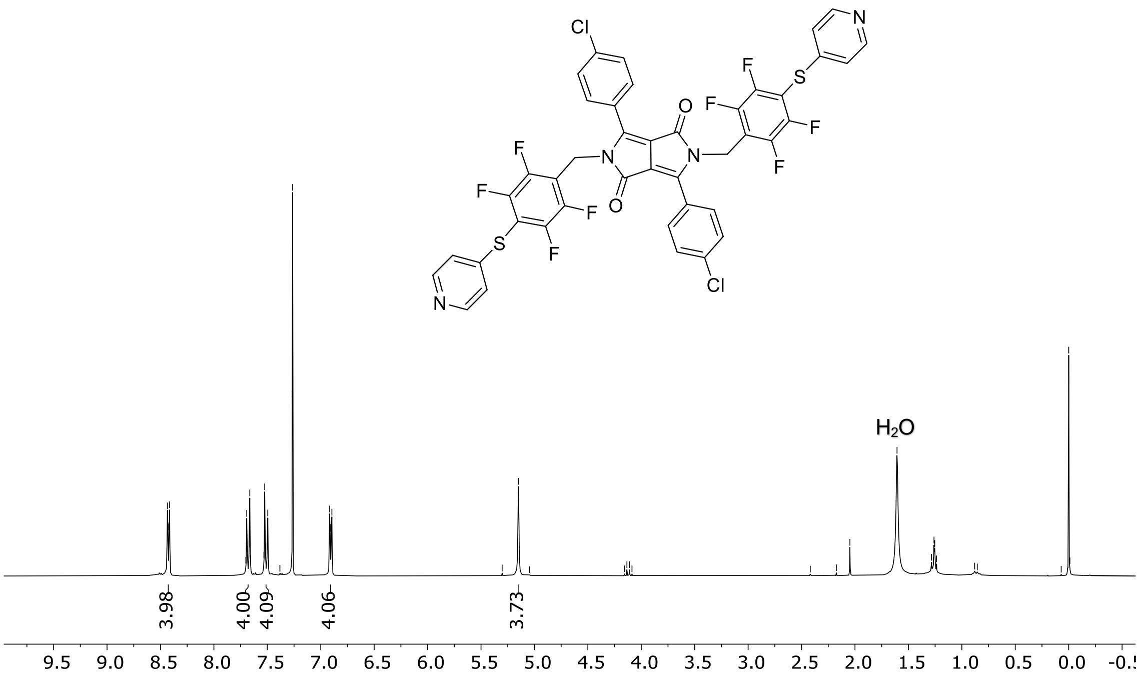

^{19}F NMR spectrum of 3a

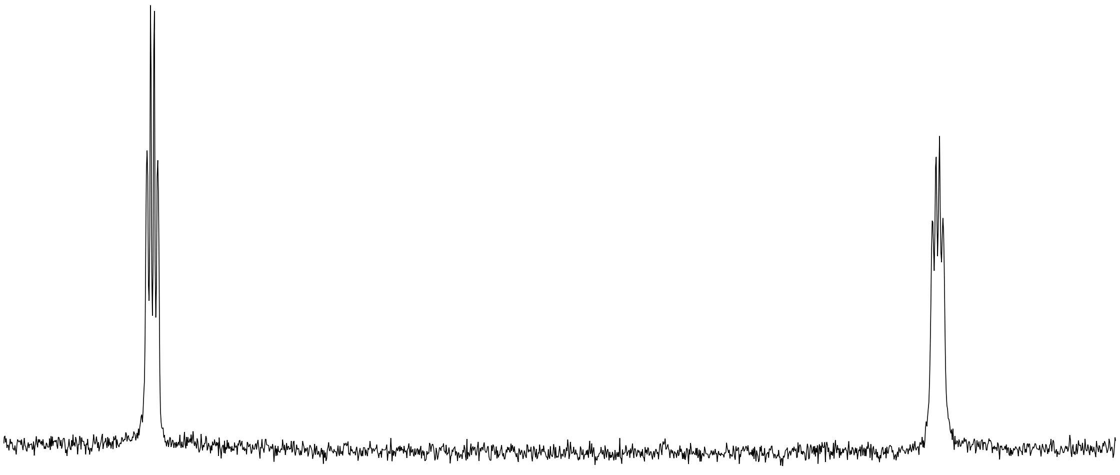

Mass spectrum of 3a

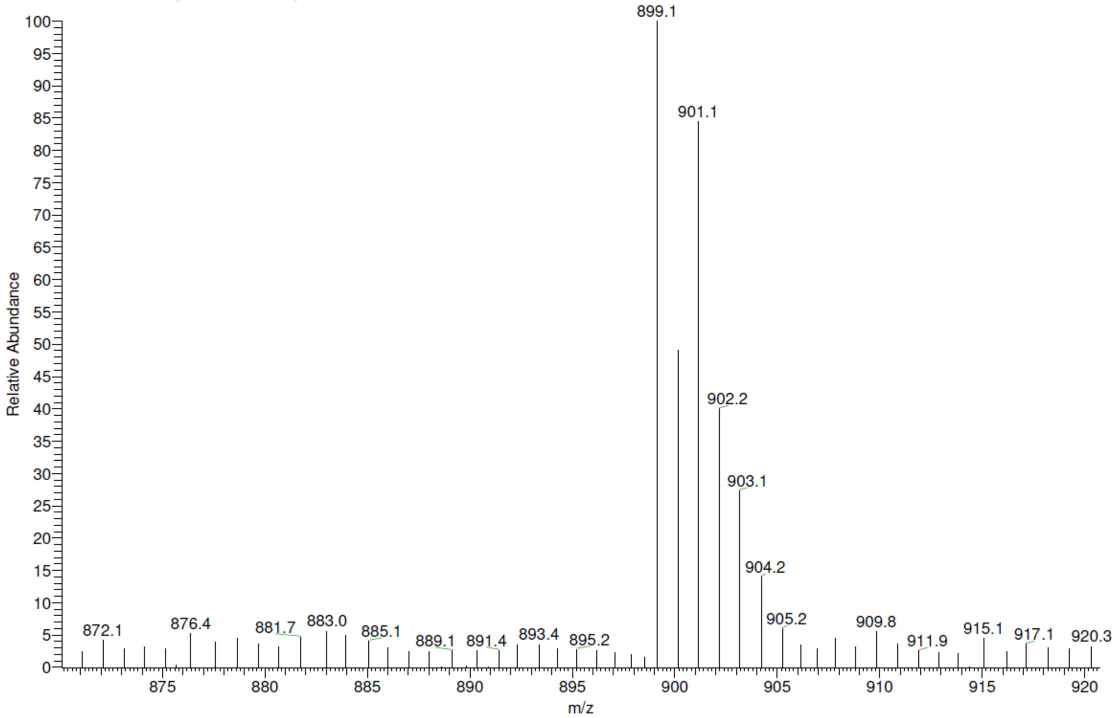

UV–vis and fluorescence spectra of 3a

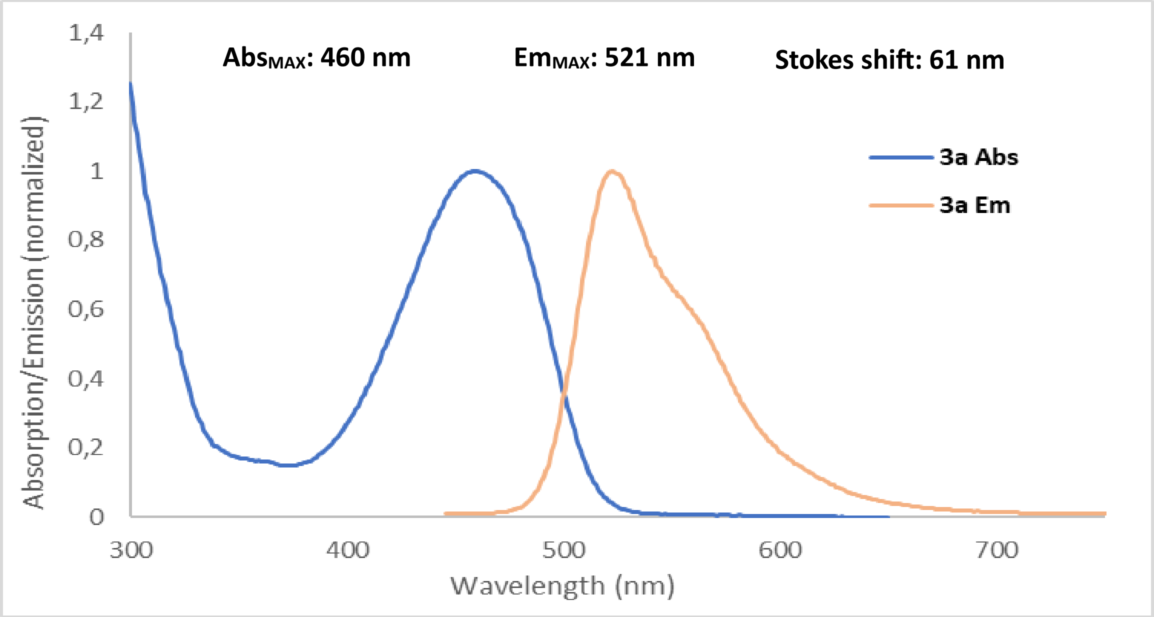

Compound 4a

^1H NMR spectrum of 4a

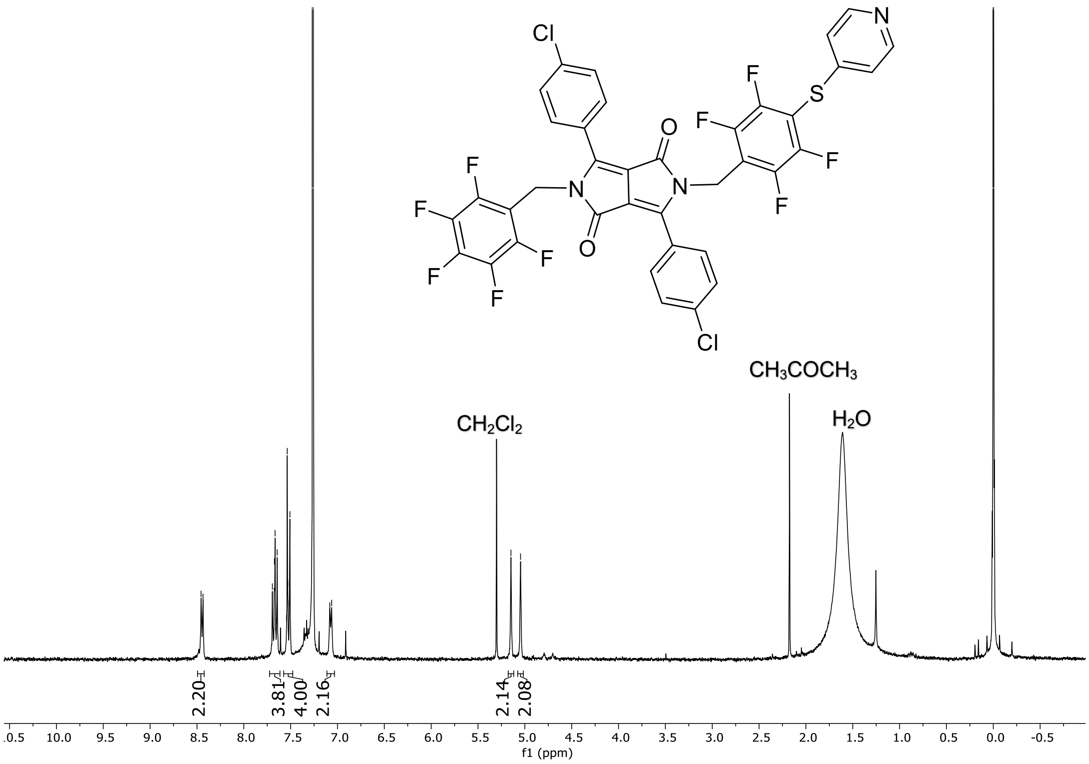

^{13}C NMR spectrum of 4a

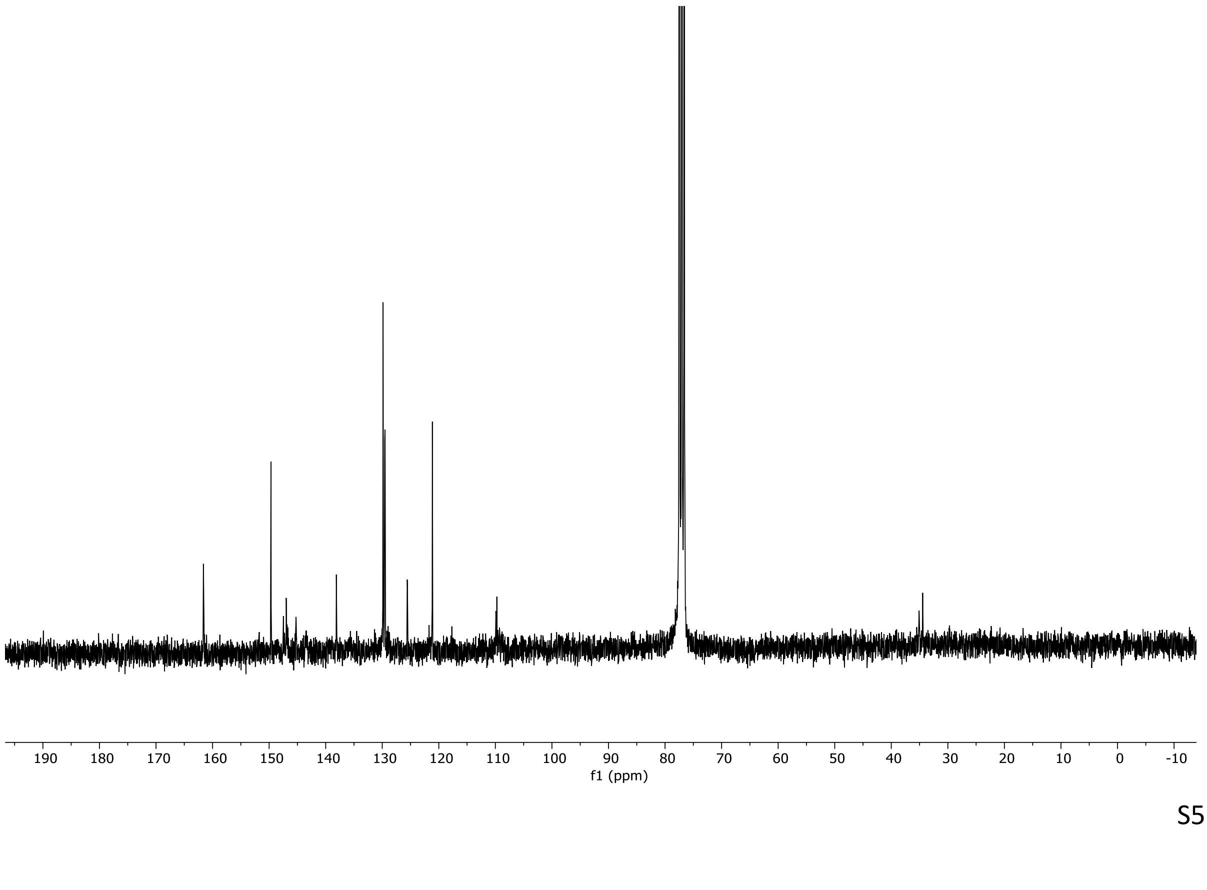

^{19}F NMR spectrum of 4a

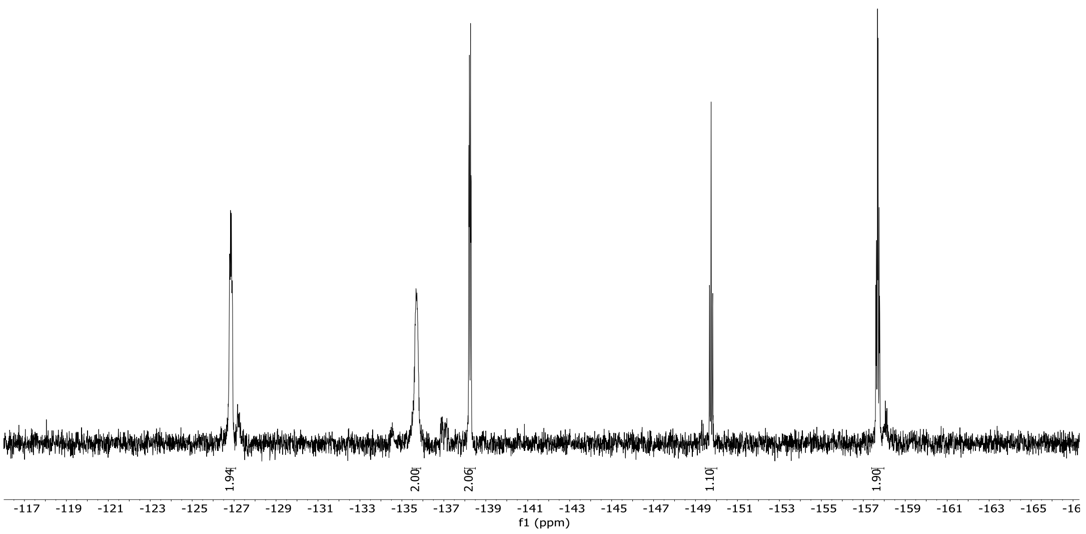

Mass spectrum of 4a

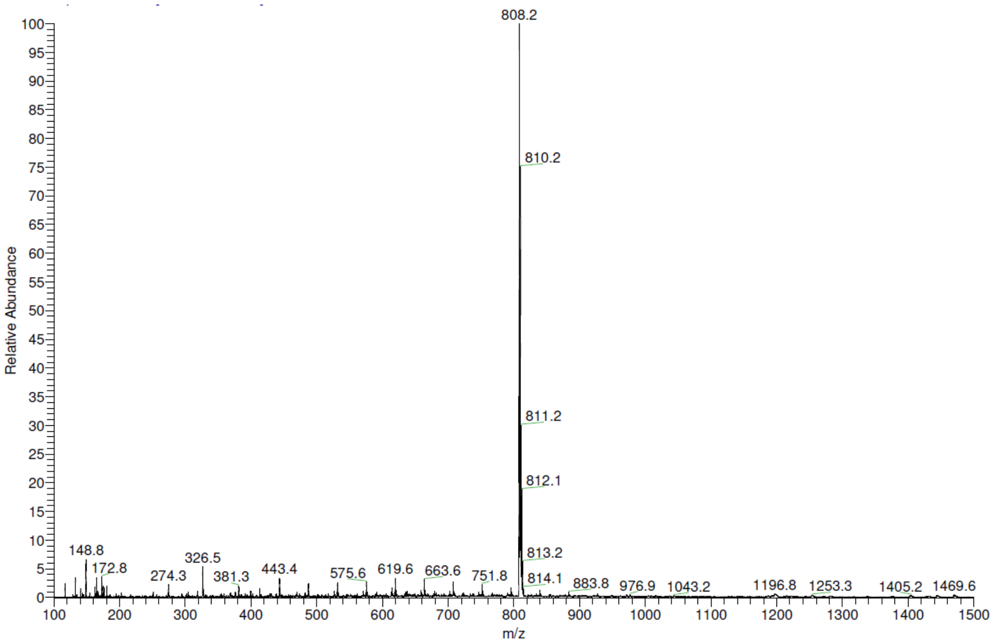

UV–vis and fluorescence spectra of 4a

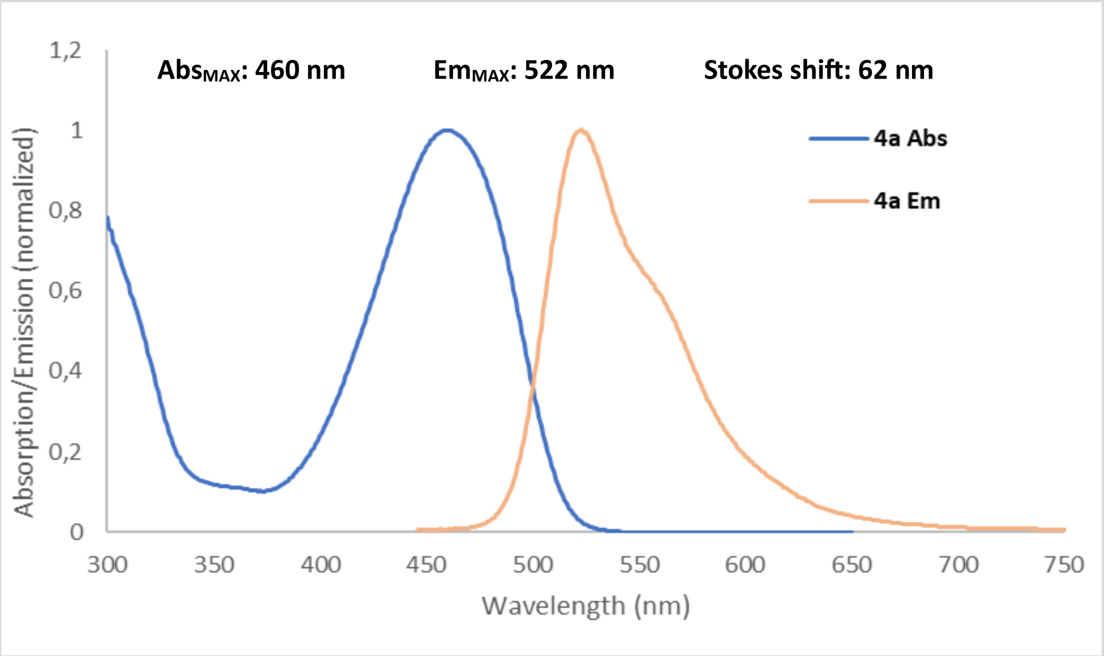

Compound 3b

^{1}H NMR spectrum of 3b

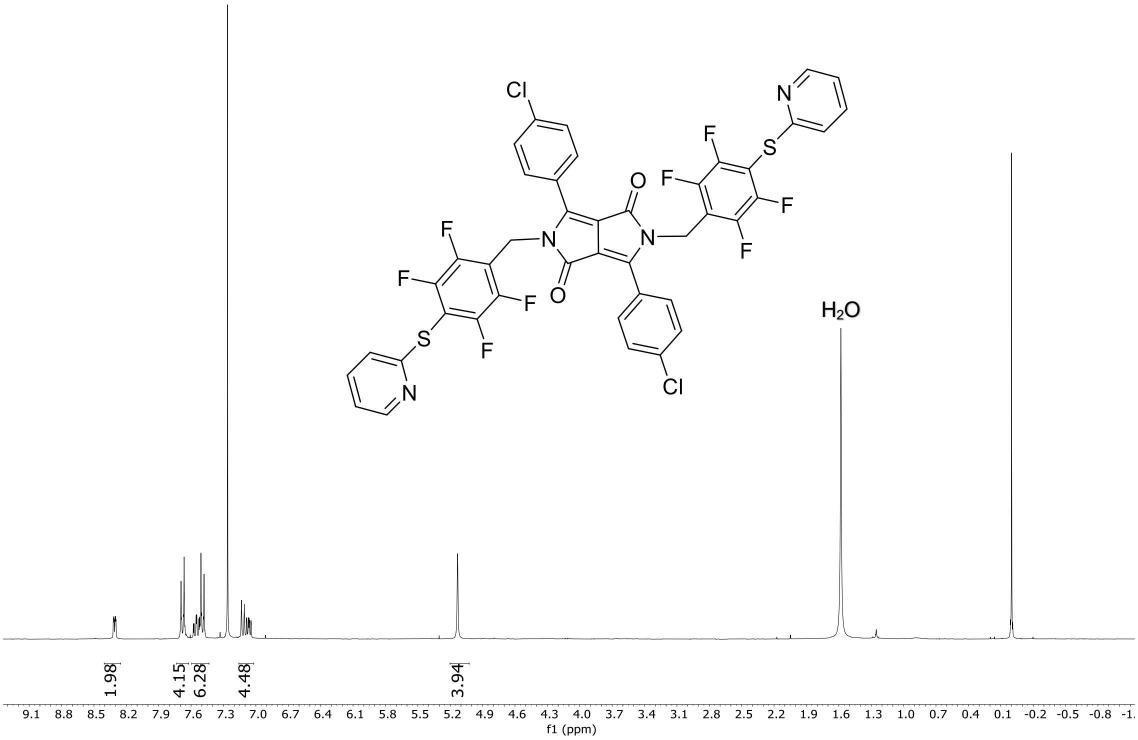

^{13}C NMR spectrum of 3b

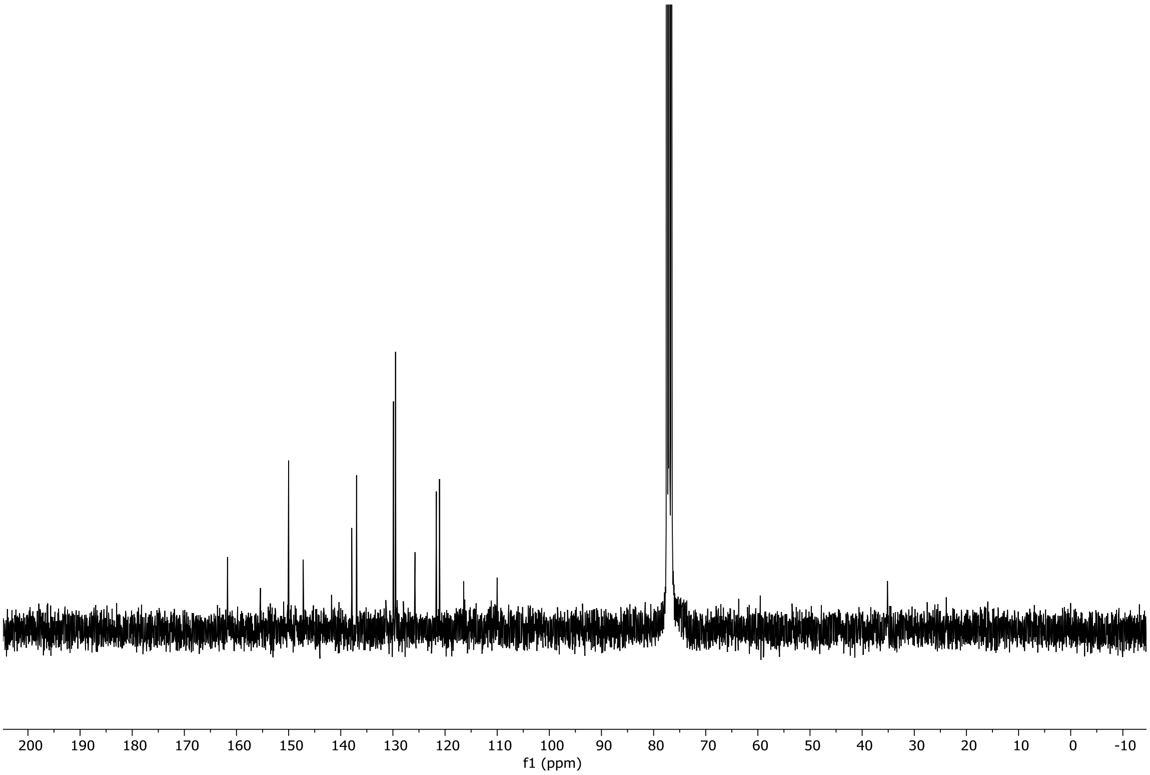

^{19}F NMR spectrum of 3b

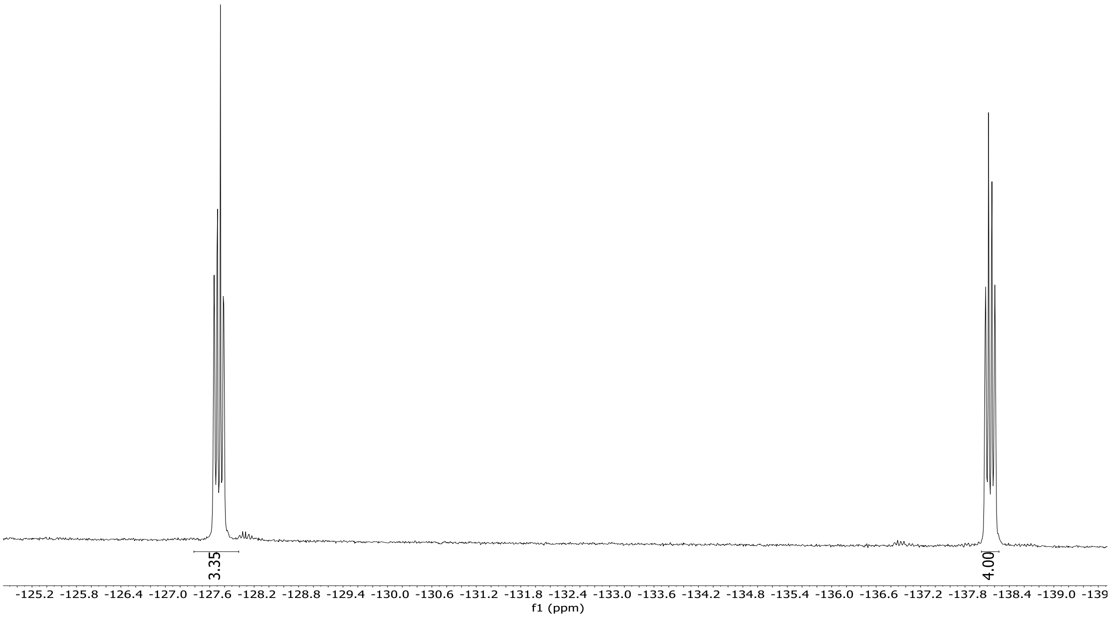

Mass spectrum of 3b

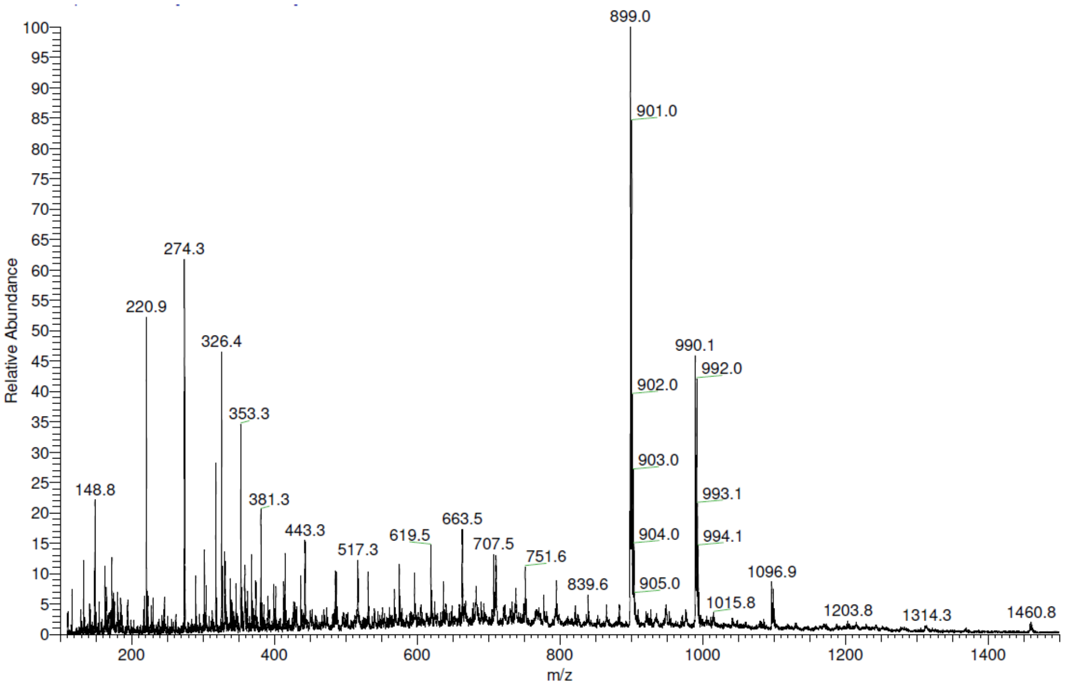

UV–vis and fluorescence spectra of 3b

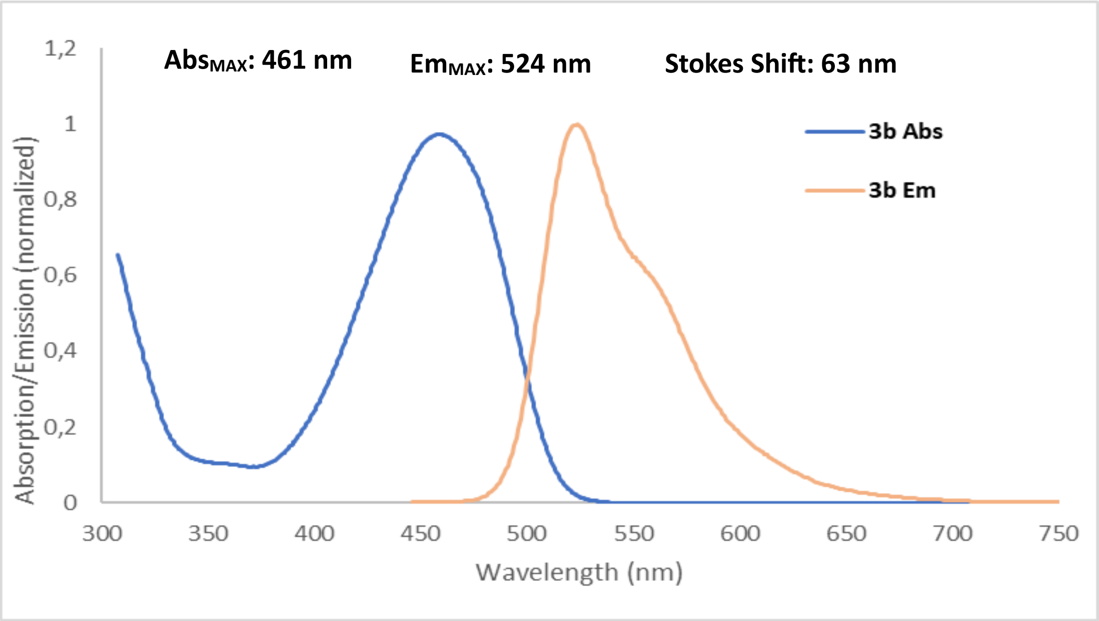

Compound 3c

^{1}H NMR spectrum of 3c

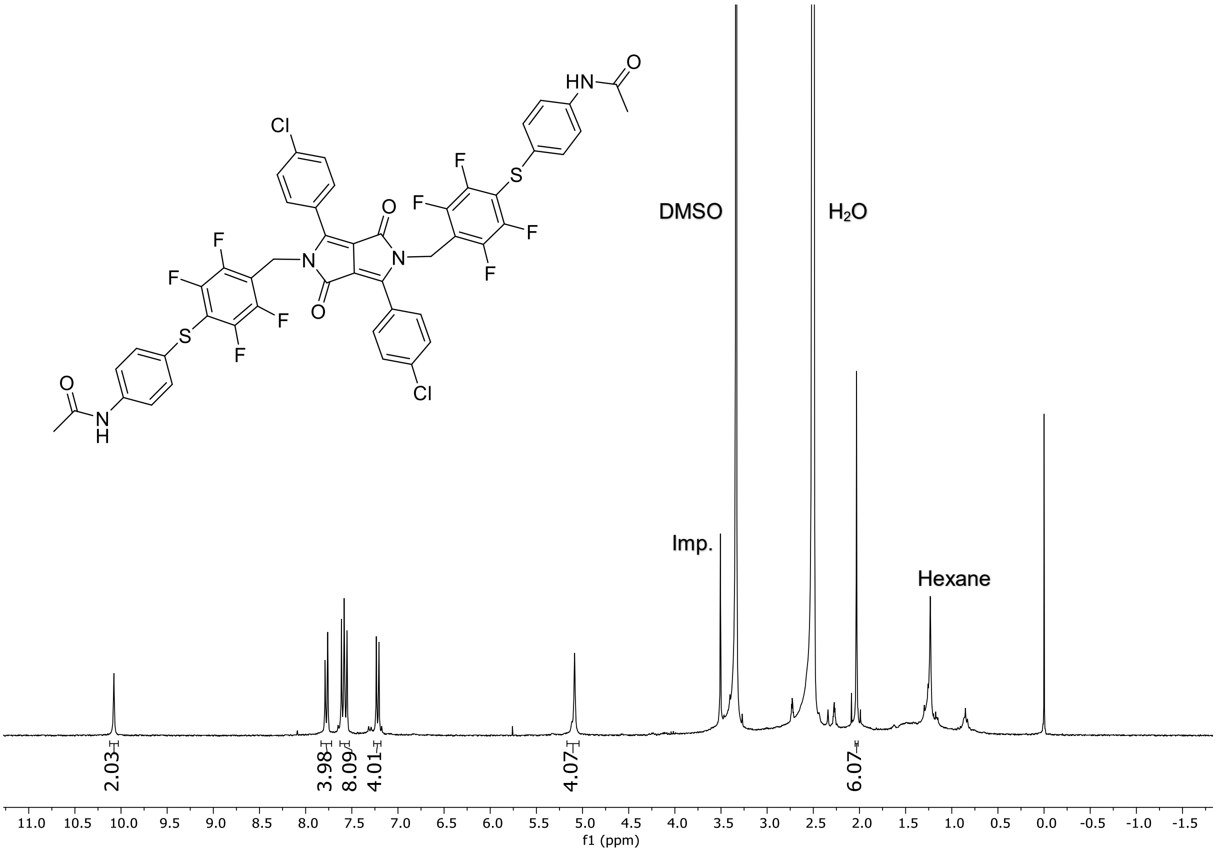

^{13}C NMR spectrum of 3c

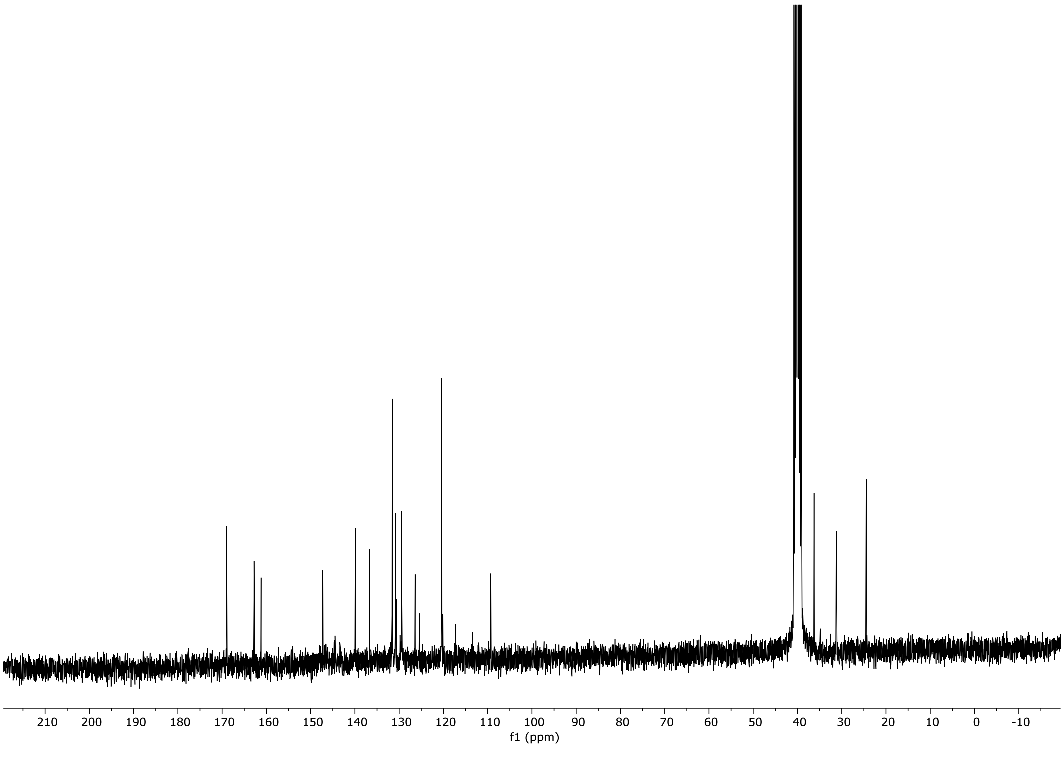

^{19}F NMR spectrum of 3c

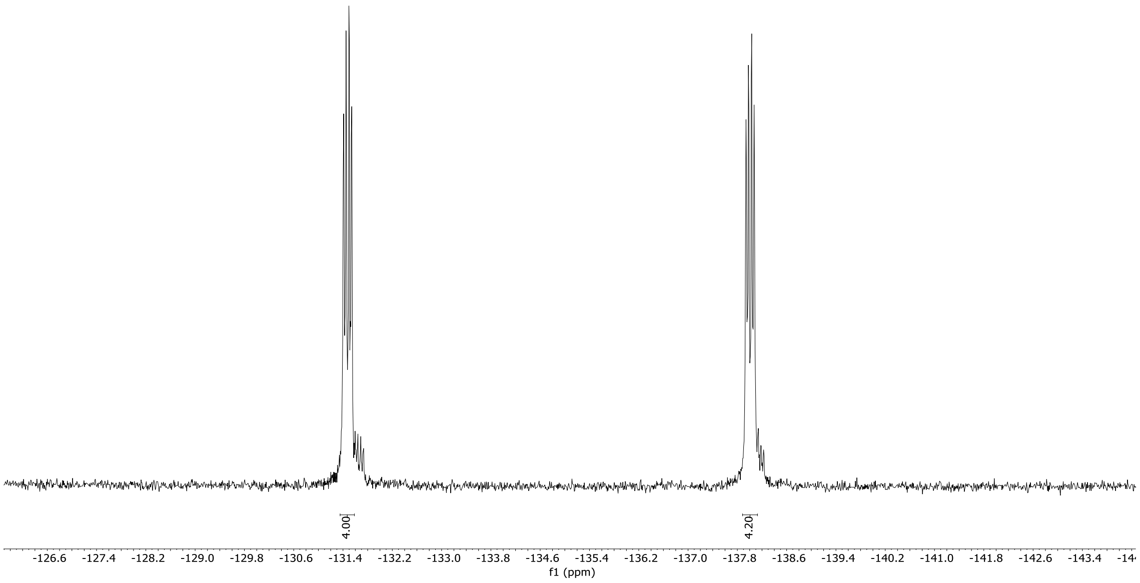

Mass spectrum of 3c

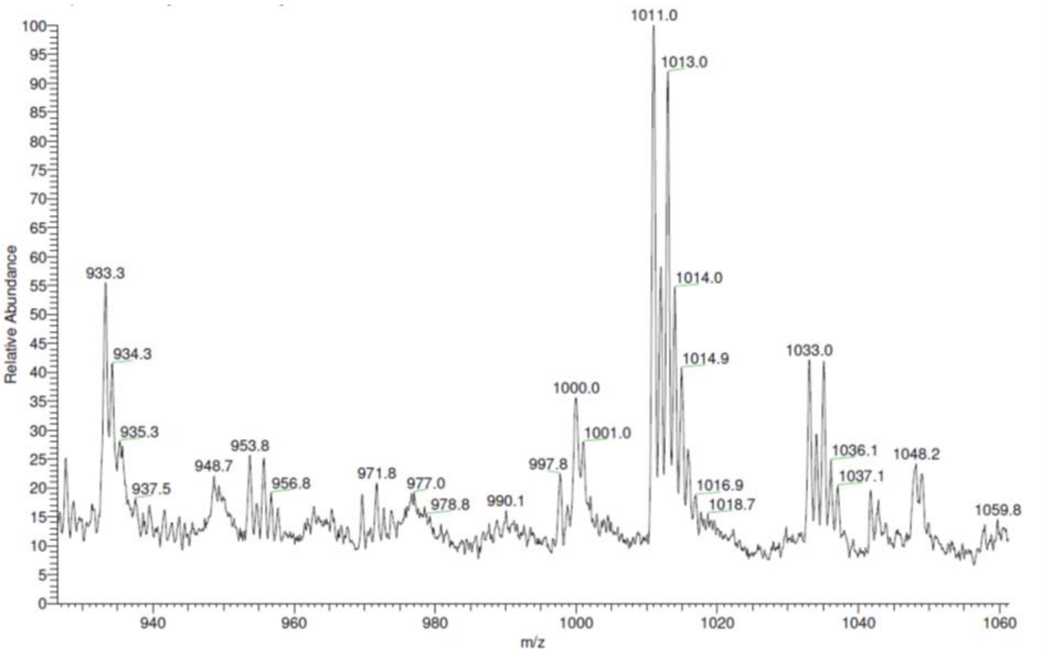

UV–vis and fluorescence spectra of 3c

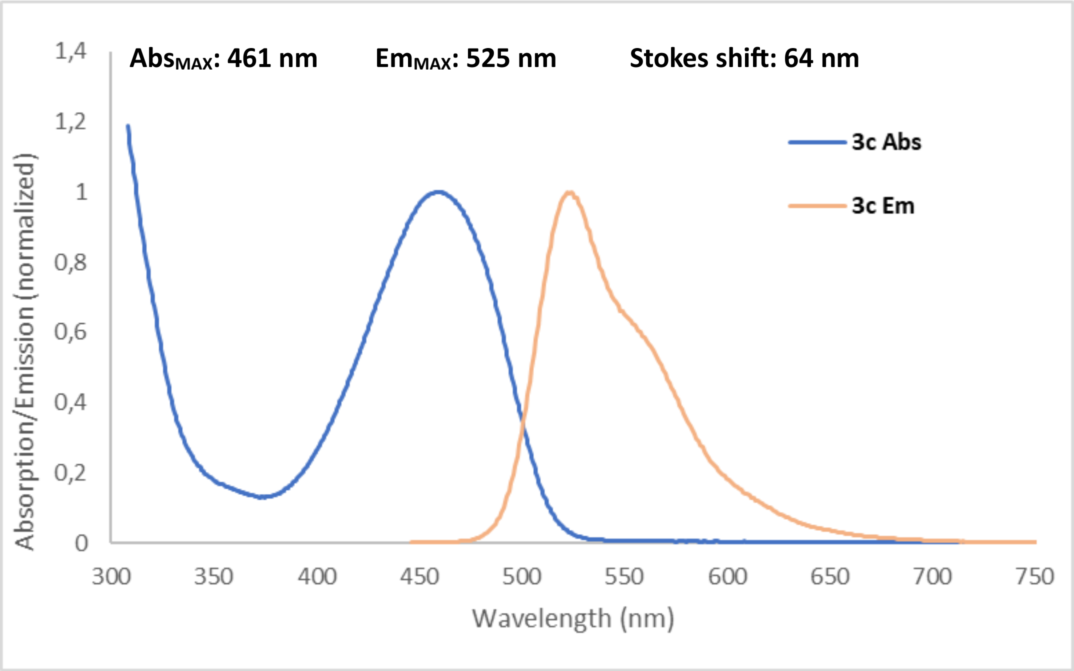

Compound 3d

^{1}H NMR spectrum of 3d

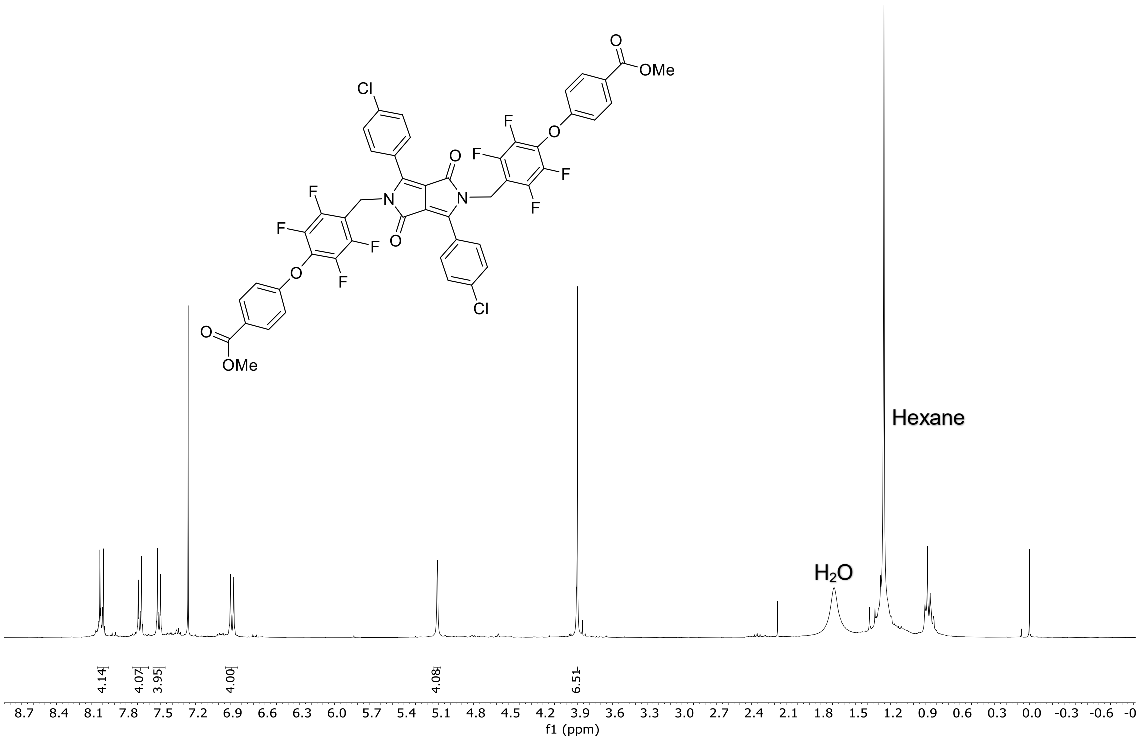

^{19}F NMR spectrum of 3d

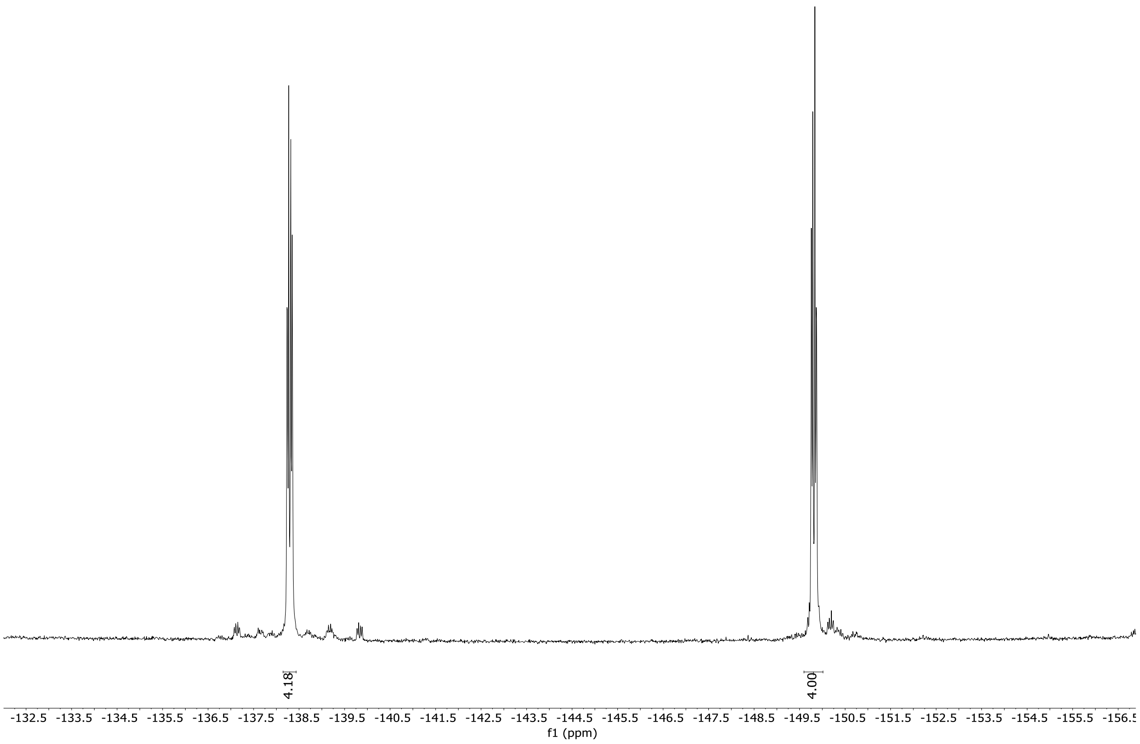

UV–vis and fluorescence spectra of 3d

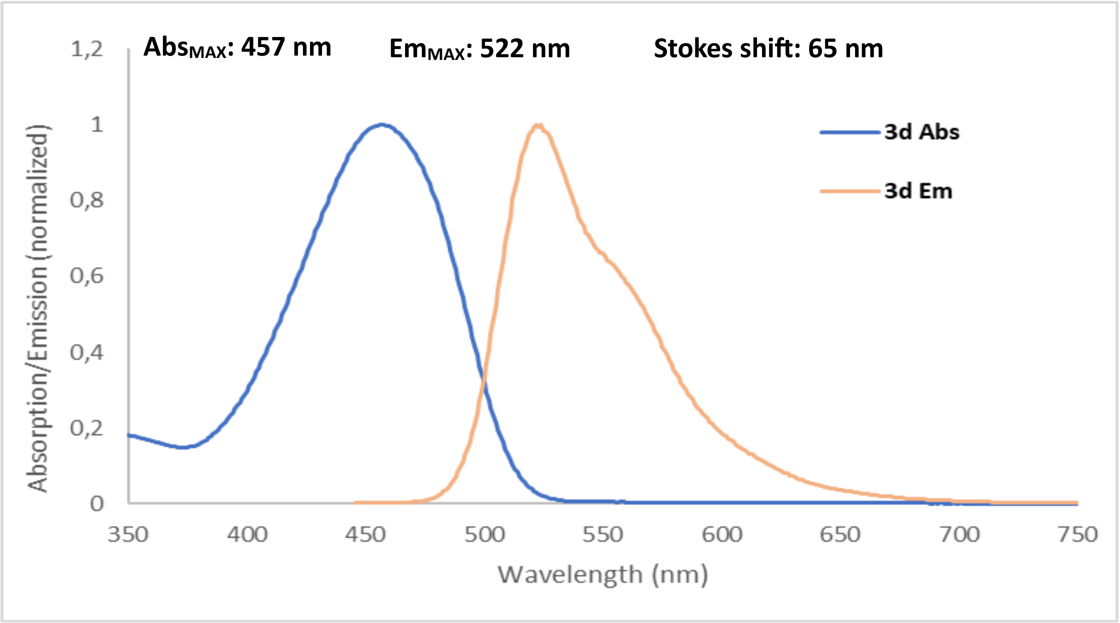

Compound 4d

¹H NMR spectrum of 4d

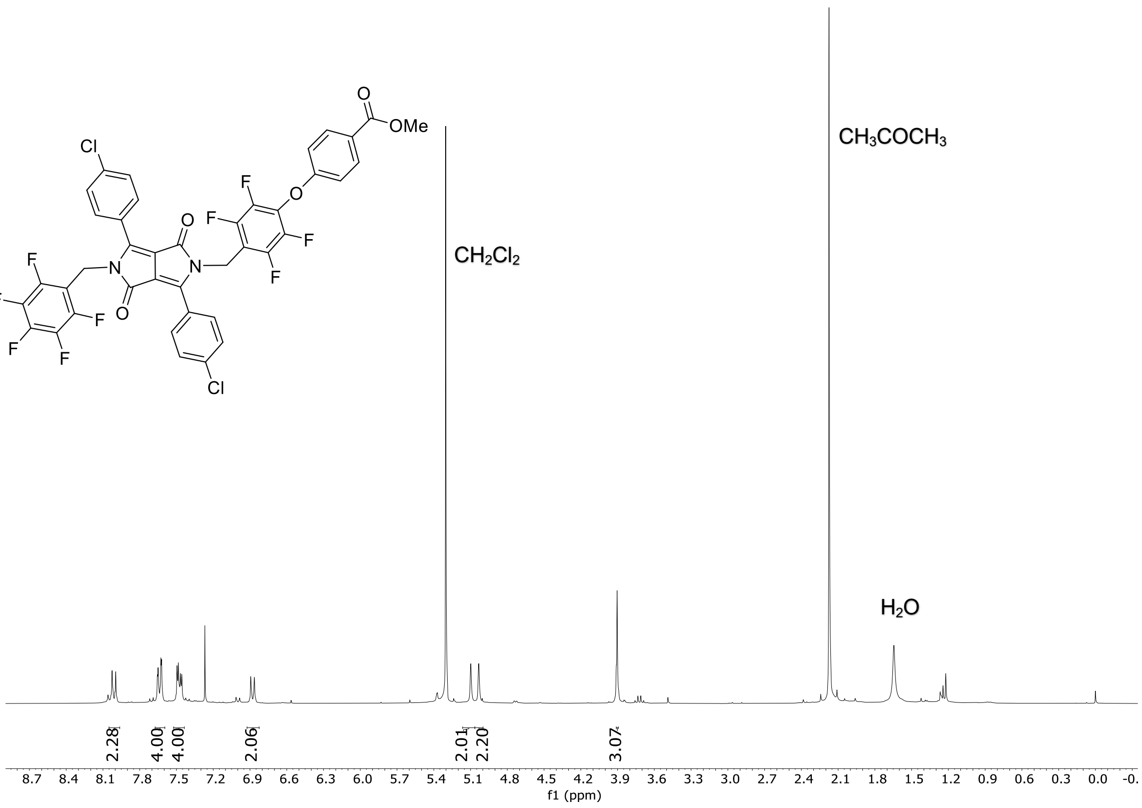

¹⁹F NMR spectrum of 4d

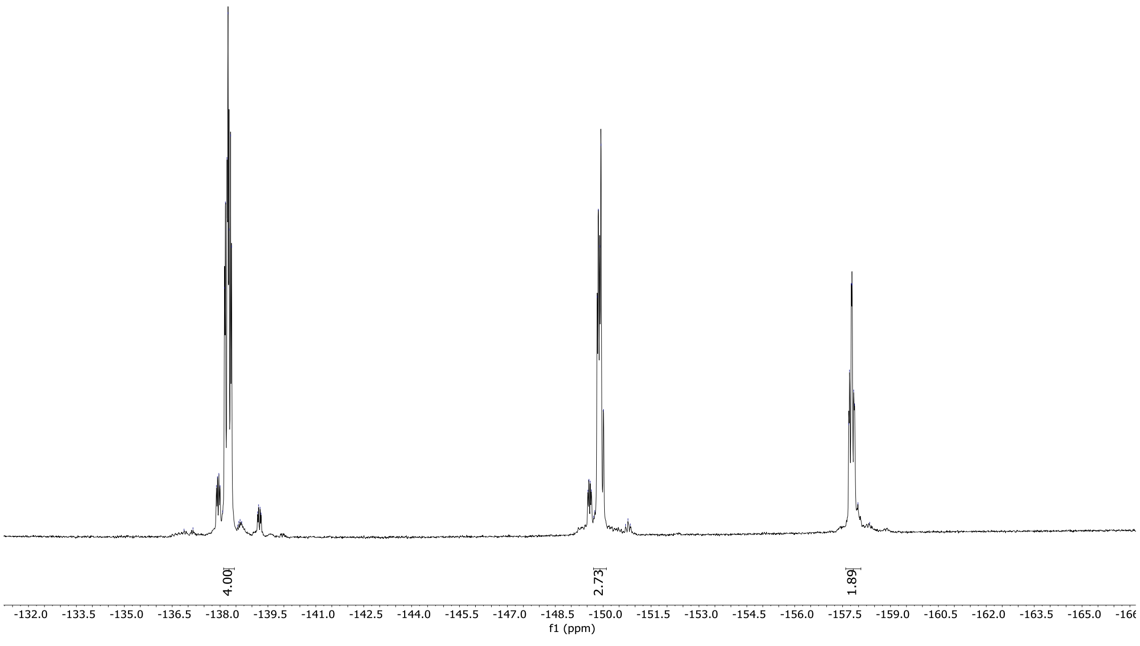

Mass spectrum of 4d

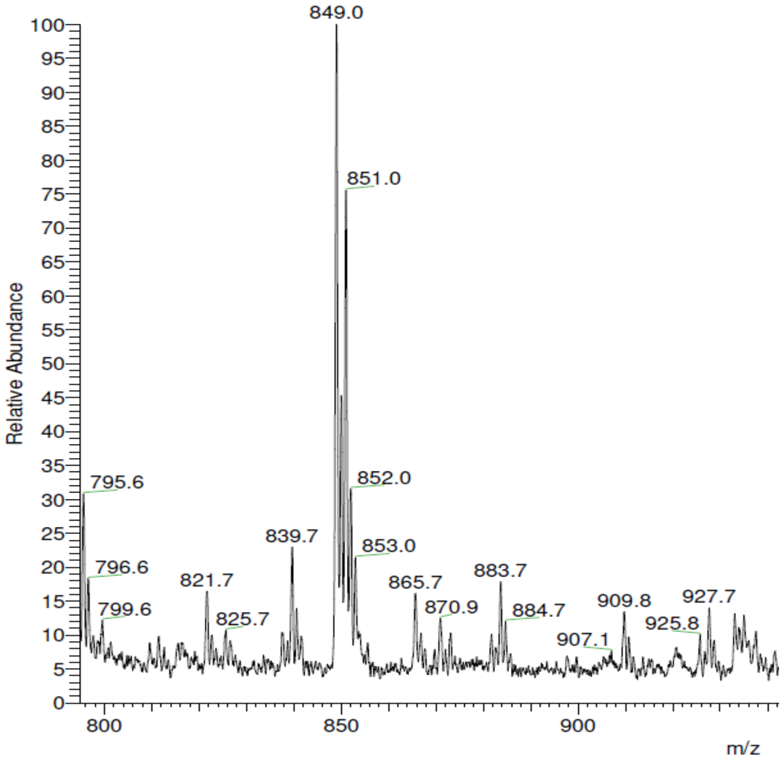

UV–vis and fluorescence spectra of 4d

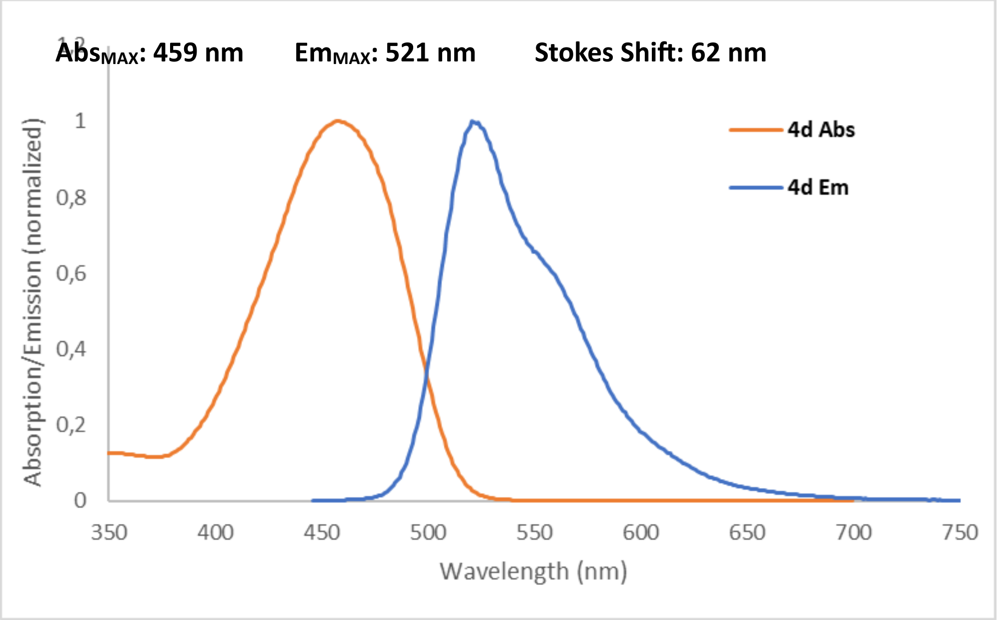

Compound 3e

^{1}H NMR spectrum of 3e

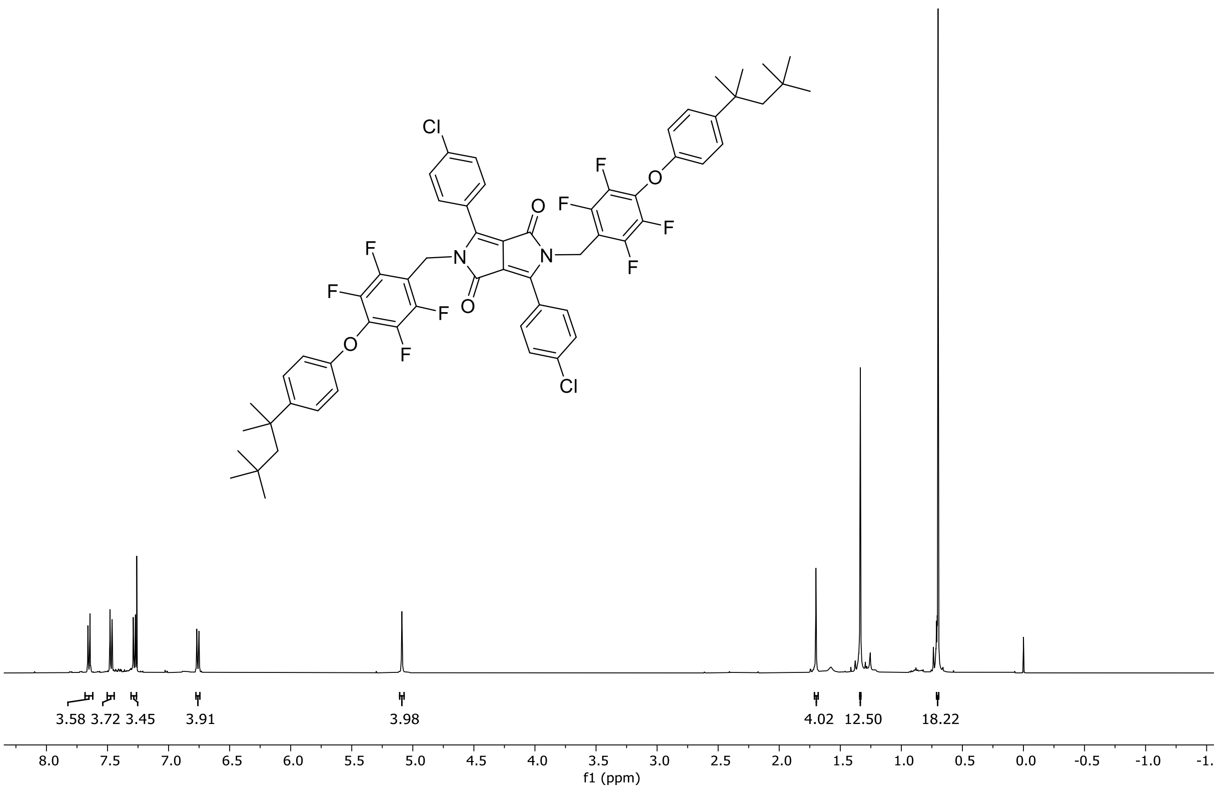

^{13}C NMR spectrum of 3e

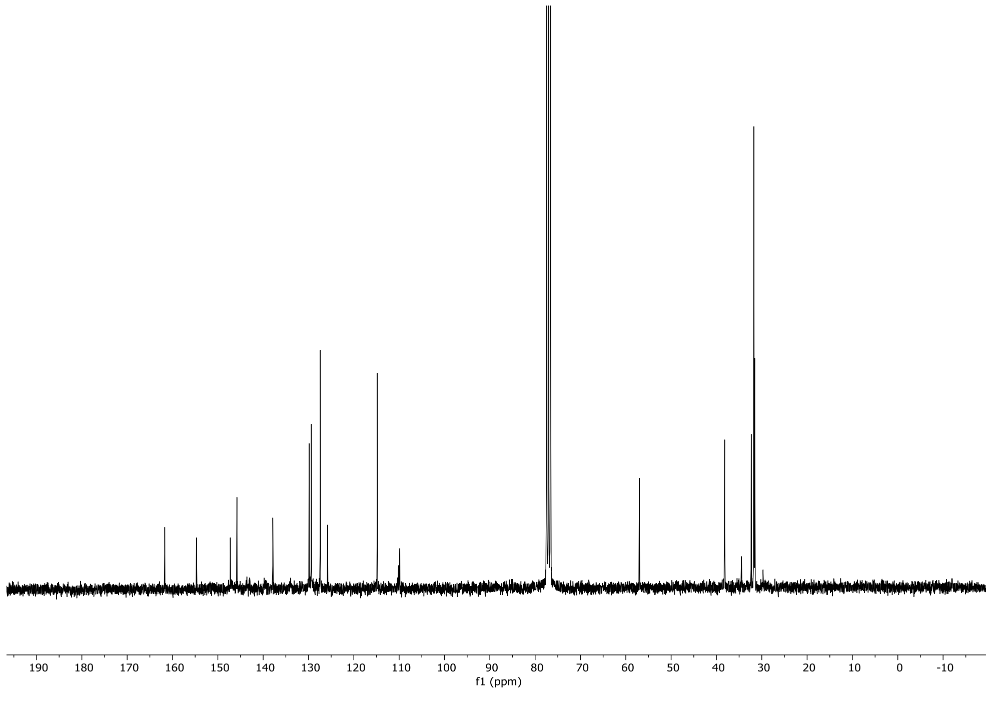

^{19}F NMR spectrum of 3e

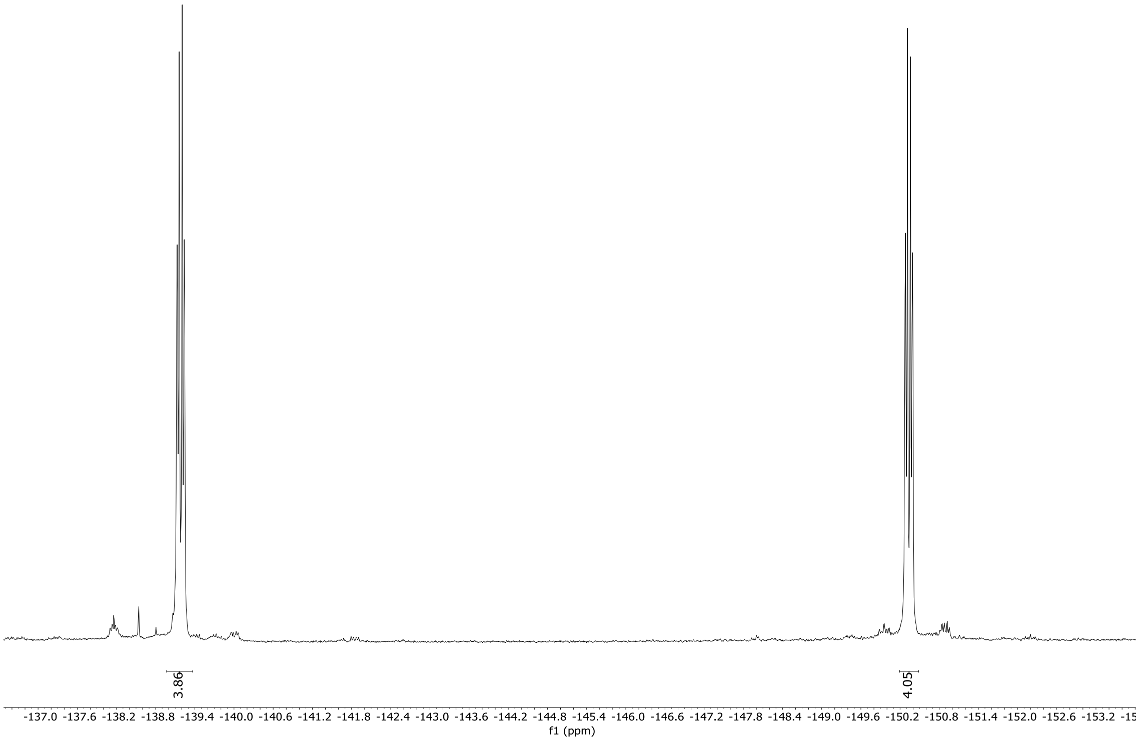

Mass spectrum of 3e

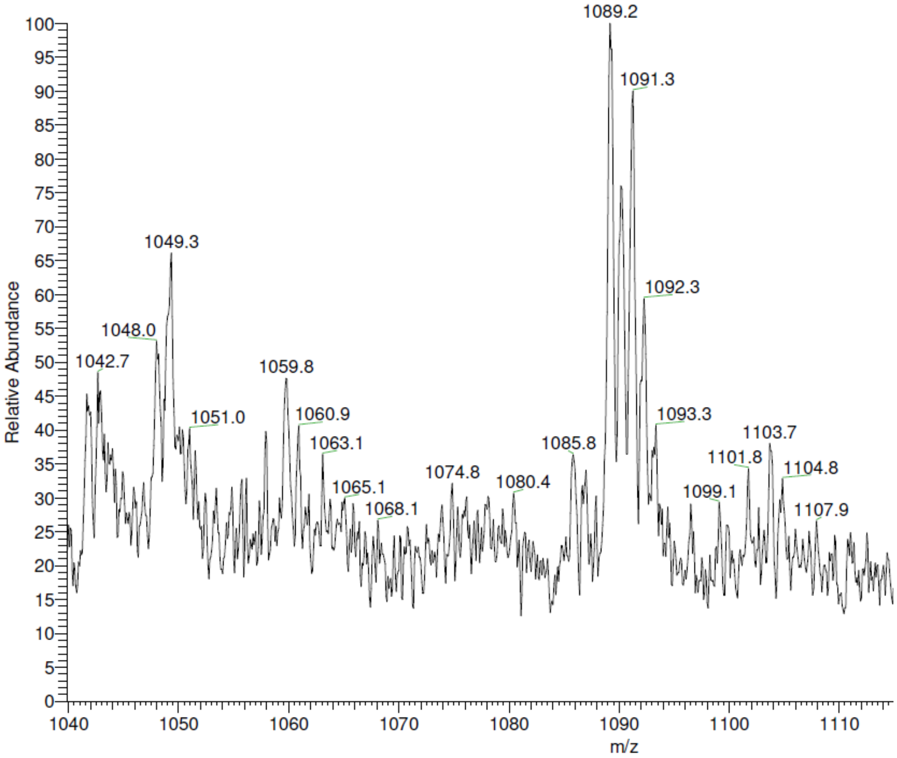

UV–vis and fluorescence spectra of 3e

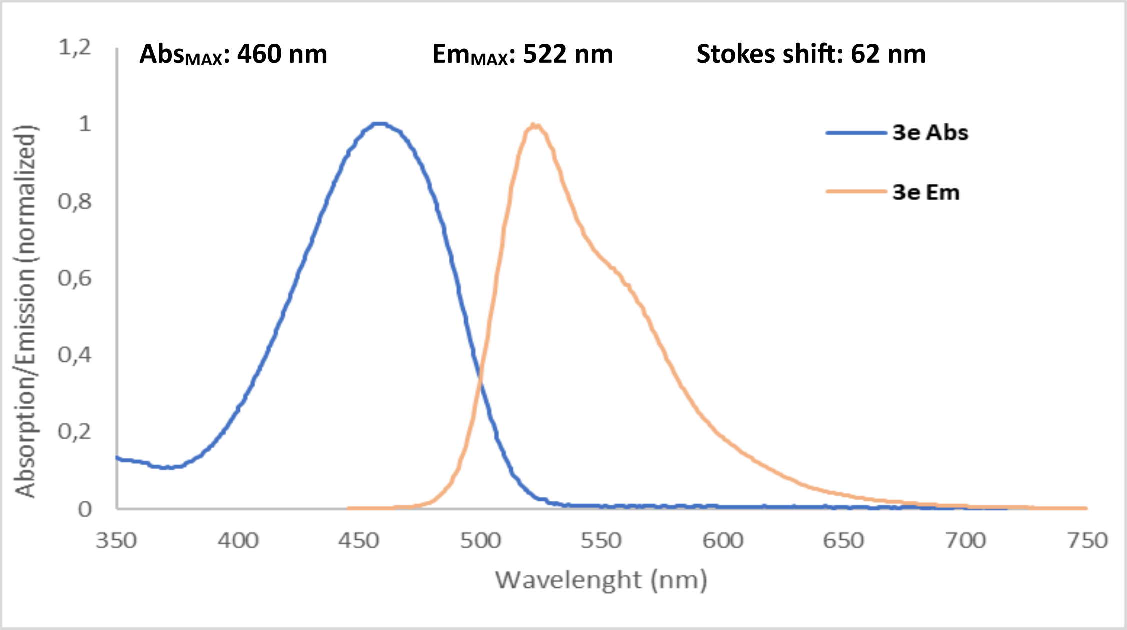

Compound 3f

^{1}H NMR spectrum of 3f

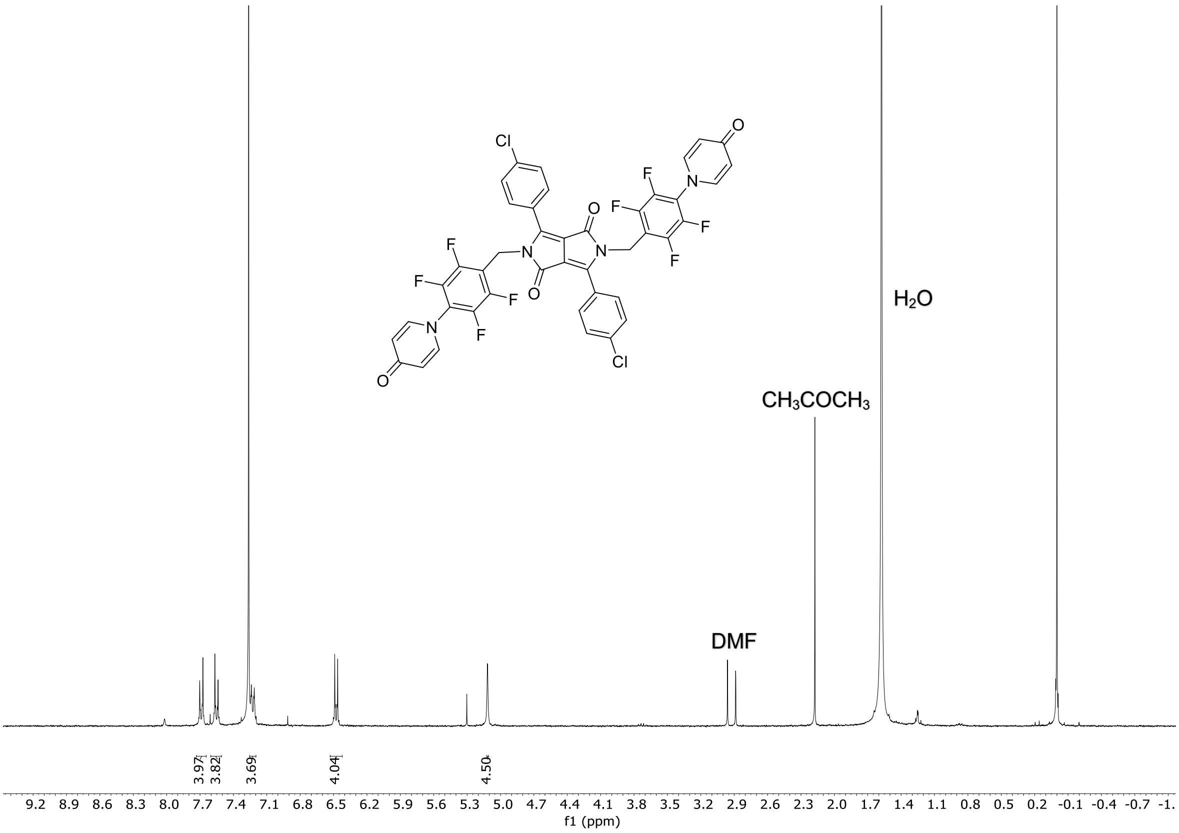

^{19}F NMR spectrum of 3f

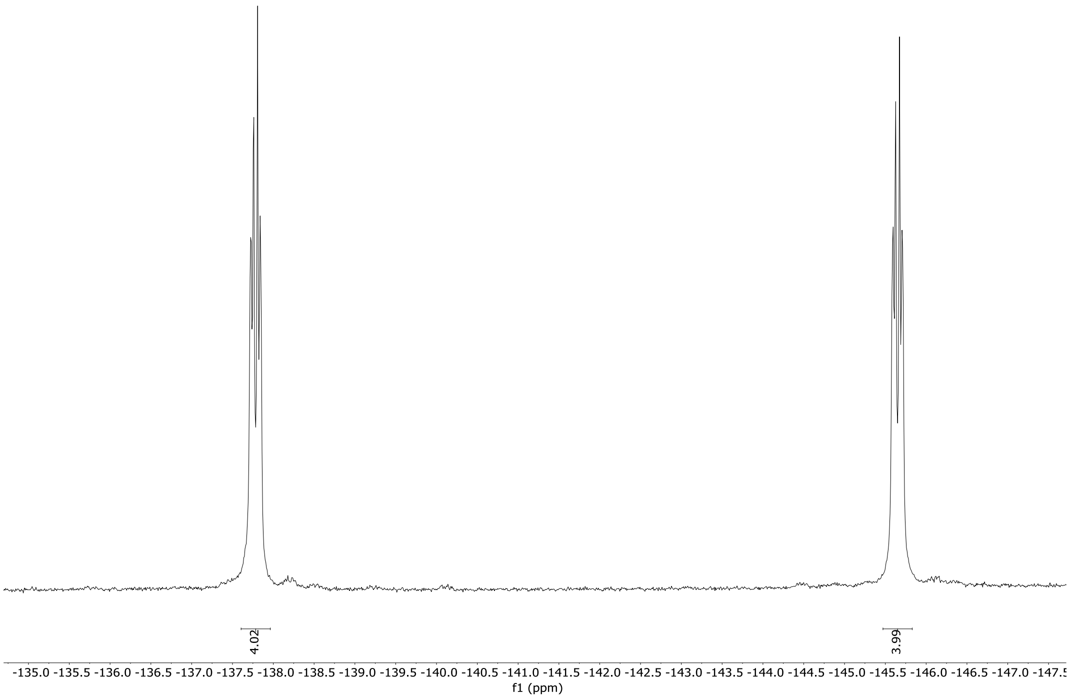

Mass spectrum of 3f

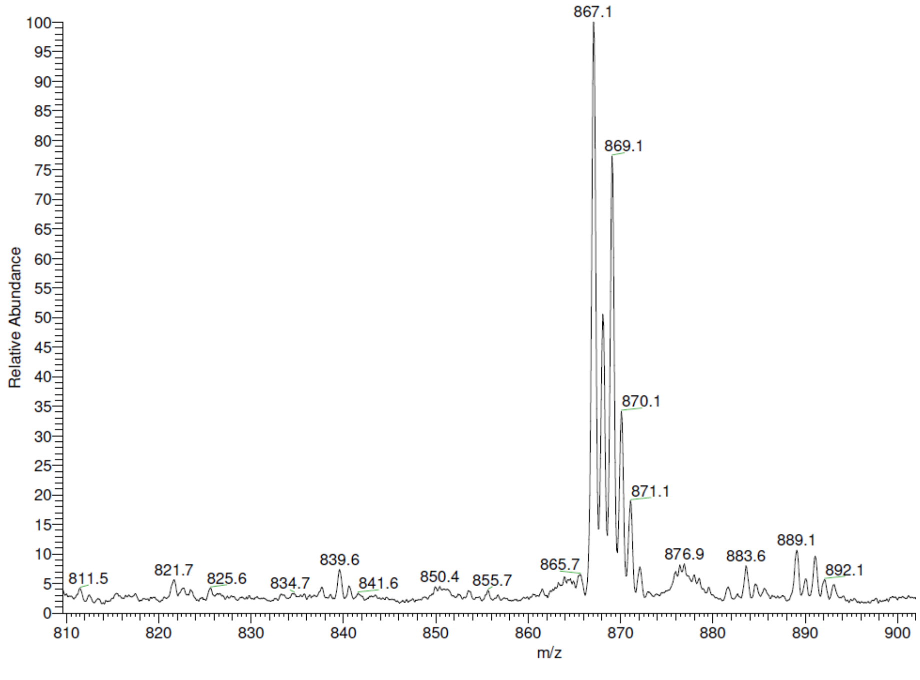

UV–vis and fluorescence spectra of 3f

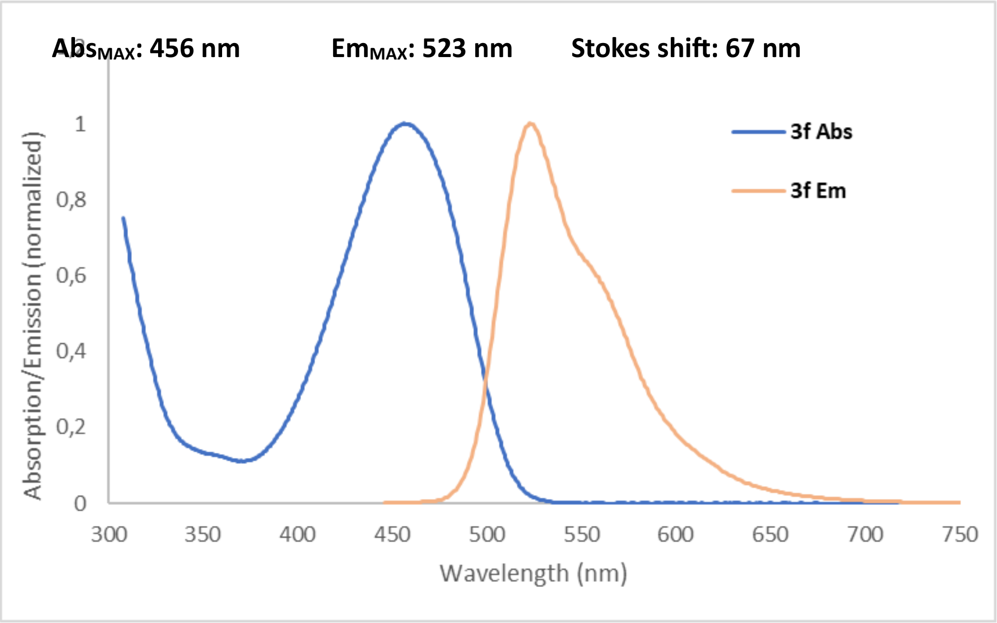

Compound 4f

^{1}H NMR spectrum of 4f

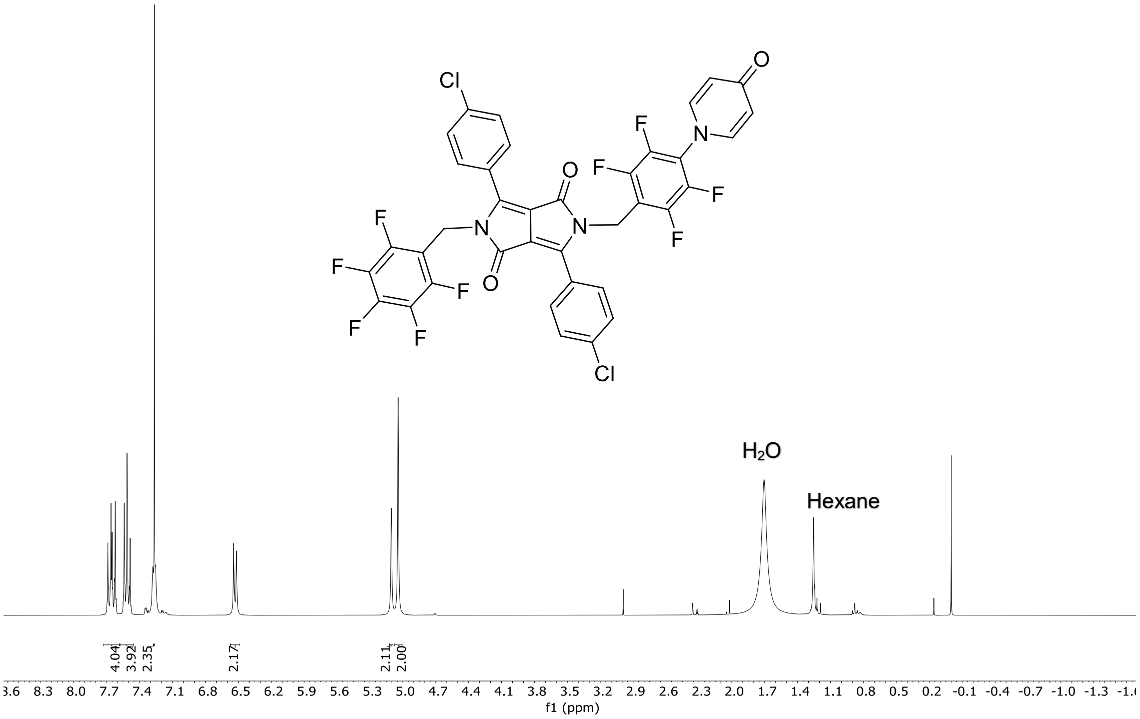

^{13}C NMR spectrum of 4f

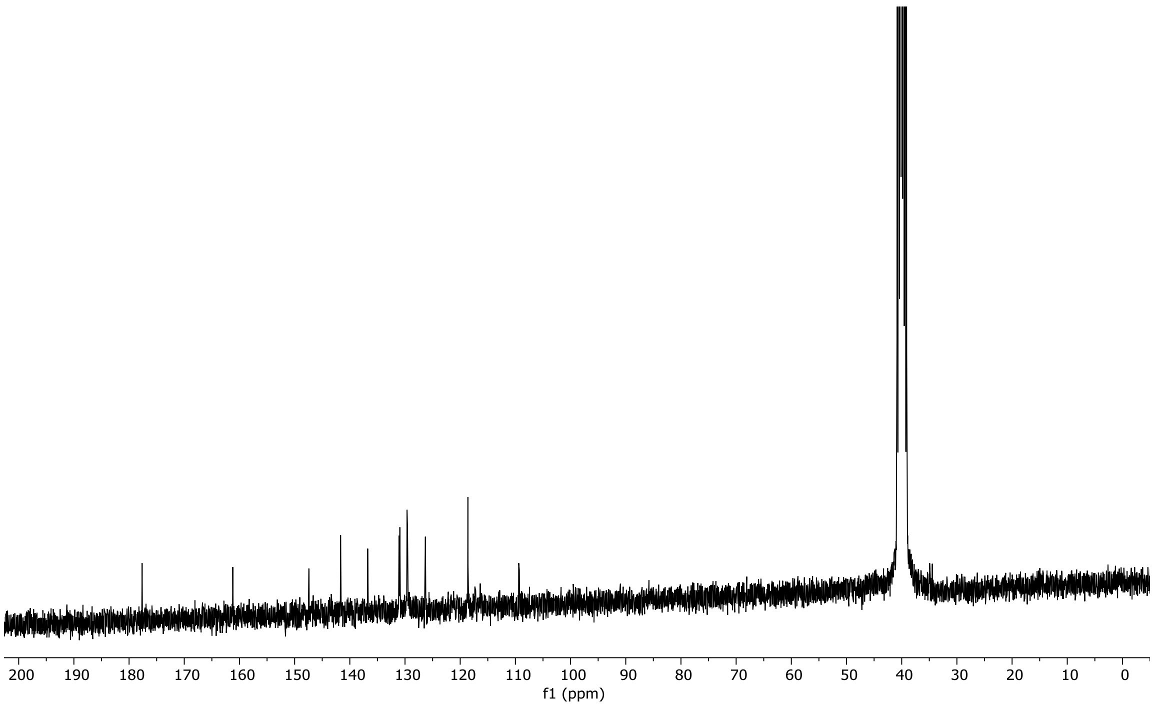

^{19}F NMR spectrum of 4f

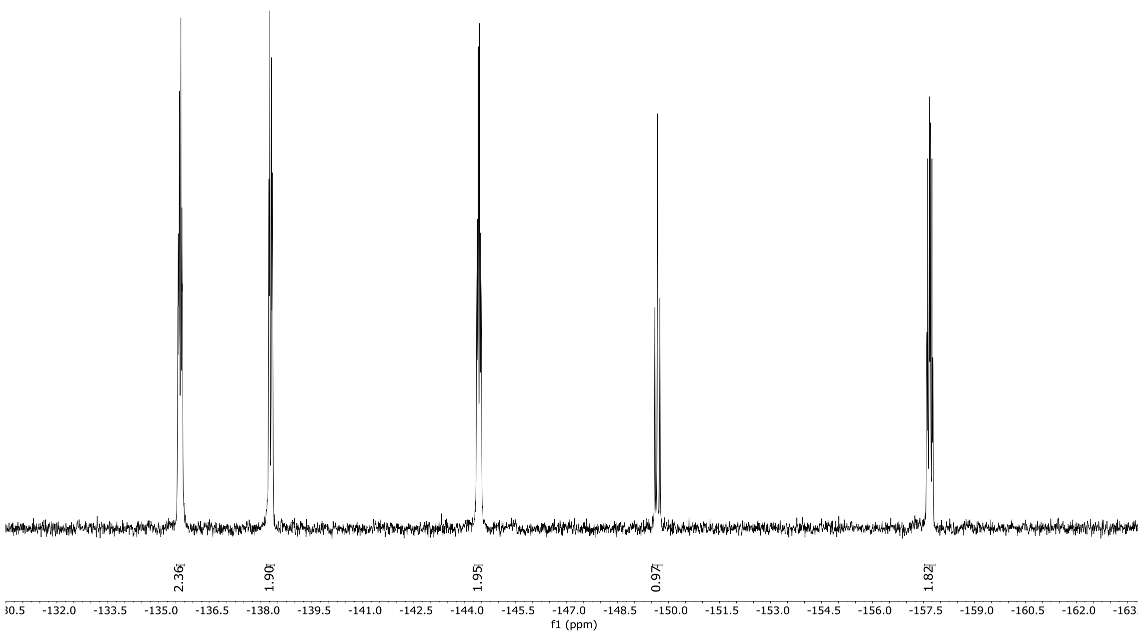

Mass spectrum of 4f

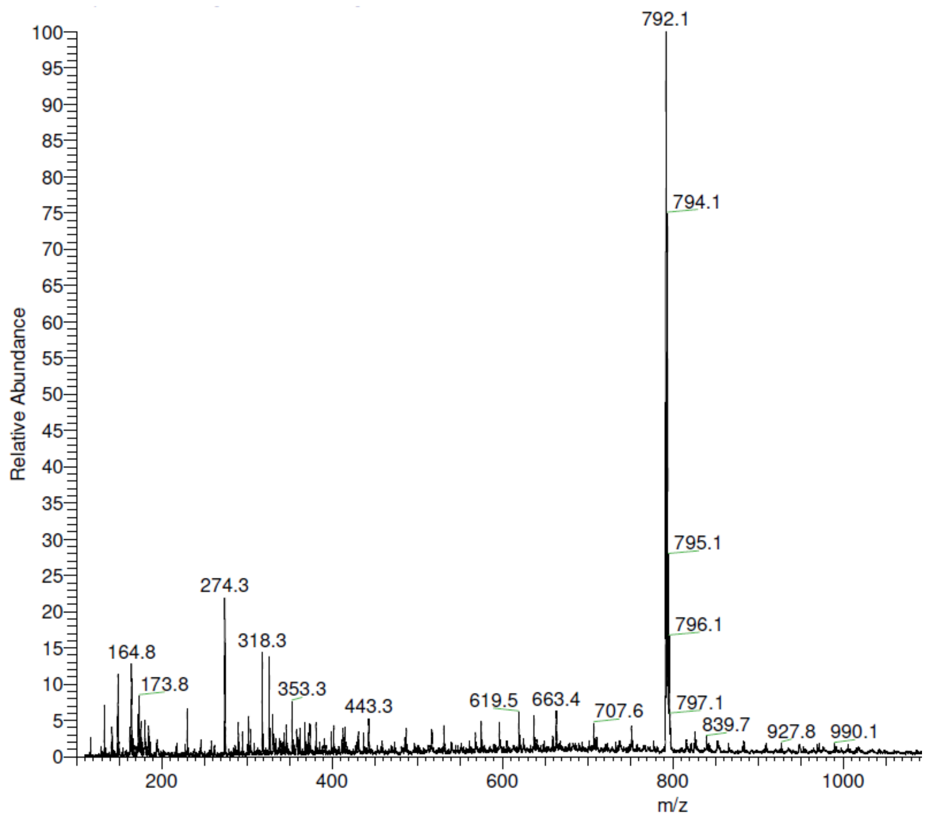

UV–vis and fluorescence spectra of 4f

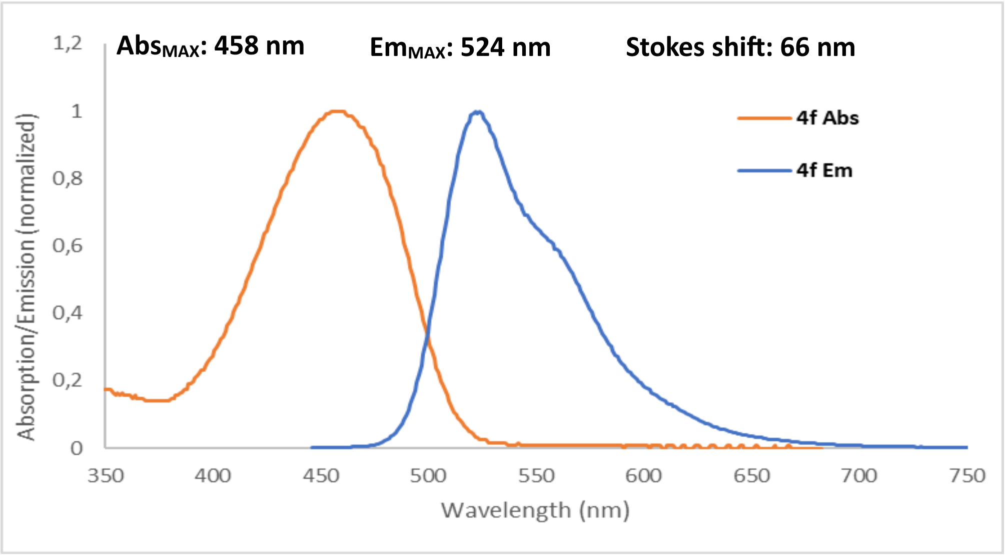

Supplement: File 1 — 1H NMR, 13C NMR and 19F NMR spectra; MS, UV–vis and emission spectra. [file Beilstein_J_Org_Chem-20-1933-s001.pdf]
